# Investigation of the Influence of Leaf Thickness on Canopy Reflectance and Physiological Traits in Upland and Pima Cotton Populations

**DOI:** 10.3389/fpls.2017.01405

**Published:** 2017-08-17

**Authors:** Duke Pauli, Jeffrey W. White, Pedro Andrade-Sanchez, Matthew M. Conley, John Heun, Kelly R. Thorp, Andrew N. French, Douglas J. Hunsaker, Elizabete Carmo-Silva, Guangyao Wang, Michael A. Gore

**Affiliations:** ^1^Plant Breeding and Genetics Section, School of Integrative Plant Science, Cornell University Ithaca, NY, United States; ^2^United States Department of Agriculture–Agricultural Research Service, Arid Land Agricultural Research Center Maricopa, AZ, United States; ^3^Department of Agricultural and Biosystems Engineering, Maricopa Agricultural Center, University of Arizona Maricopa, AZ, United States; ^4^Maricopa Agricultural Center, School of Plant Sciences, University of Arizona Maricopa, AZ, United States

**Keywords:** abiotic stress, leaf thickness, canopy reflectance, cotton, high-throughput phenotyping, specific leaf weight

## Abstract

Many systems for field-based, high-throughput phenotyping (FB-HTP) quantify and characterize the reflected radiation from the crop canopy to derive phenotypes, as well as infer plant function and health status. However, given the technology's nascent status, it remains unknown how biophysical and physiological properties of the plant canopy impact downstream interpretation and application of canopy reflectance data. In that light, we assessed relationships between leaf thickness and several canopy-associated traits, including normalized difference vegetation index (NDVI), which was collected via active reflectance sensors carried on a mobile FB-HTP system, carbon isotope discrimination (CID), and chlorophyll content. To investigate the relationships among traits, two distinct cotton populations, an upland (*Gossypium hirsutum* L.) recombinant inbred line (RIL) population of 95 lines and a Pima (*G. barbadense* L.) population composed of 25 diverse cultivars, were evaluated under contrasting irrigation regimes, water-limited (WL) and well-watered (WW) conditions, across 3 years. We detected four quantitative trait loci (QTL) and significant variation in both populations for leaf thickness among genotypes as well as high estimates of broad-sense heritability (on average, above 0.7 for both populations), indicating a strong genetic basis for leaf thickness. Strong phenotypic correlations (maximum *r* = −0.73) were observed between leaf thickness and NDVI in the Pima population, but not the RIL population. Additionally, estimated genotypic correlations within the RIL population for leaf thickness with CID, chlorophyll content, and nitrogen discrimination (${\hat{r}}_{gij}$ = −0.32, 0.48, and 0.40, respectively) were all significant under WW but not WL conditions. Economically important fiber quality traits did not exhibit significant phenotypic or genotypic correlations with canopy traits. Overall, our results support considering variation in leaf thickness as a potential contributing factor to variation in NDVI or other canopy traits measured via proximal sensing, and as a trait that impacts fundamental physiological responses of plants.

## Introduction

Field-based high-throughput phenotyping (HTP) offers the potential of rapidly and accurately characterizing phenotypic variation in large populations grown under conditions that are relevant to commercial crop production (reviewed in White et al., 2012; Pauli et al., 2016b). Most methods proposed for HTP under field conditions employ measurements of reflected radiation or thermal emissions from the crop canopy. For such measurements, the uppermost leaves in the canopy are usually the dominant visible component, unless reproductive organs have emerged above the foliage, with which light interacts. In characterizing crop traits via proximal sensing methods using instruments mounted on high-clearance tractors or unmanned aerial vehicles, it is important to understand how variation in leaf traits affect canopy reflectance. One such trait that is of particular importance is the physical thickness of a leaf.

Leaf thickness largely determines the length of the optical path of light through a leaf and the number of anatomical features (e.g., cell walls and chloroplasts) that either reflect, absorb, or transmit light. The trait also has important relationships with biomass partitioning, net productivity and crop response to water deficits. A fundamental tradeoff exists between partitioning strategies that favor thinner leaves with a greater leaf surface area per unit leaf mass, as opposed to thicker leaves and less leaf area (Poorter and Remkes, 1990). While greater surface area has the potential to increase light interception, thicker leaves typically have greater photosynthetic rates (Pettigrew et al., 1993). Water deficits are often associated with leaf thickness and otherwise affect traits associated with leaf thickness such as leaf water content, osmotic potential, and transpiration, which may relate to compensation for reduced expansion of leaf surfaces (area).

Leaf area index (LAI, the total leaf area per unit area of land) can be expressed as the product of leaf mass per unit land area (L) and the specific leaf area (SLA), where the SLA is the ratio of leaf area to leaf mass (fresh or dry). To provide a more direct association with leaf thickness, the inverse of SLA, the specific leaf weight (SLW) is used, and we subsequently emphasize SLW. Although the relation of physical thickness to SLW is somewhat complicated by variation in water content and in the volume of gas-filled space in the mesophyll, leaf thickness usually varies proportionally with SLW. Also, SLW often is proportional to concentrations of chlorophyll and total leaf nitrogen when concentration is expressed on a leaf area basis (White and Montes, 2005).

Leidi et al. (1999) detected large variation in SLW of cotton and also found that SLW decreased with transpiration efficiency, measured as carbon isotope discrimination (CID) and seed cotton yield. Given the evidence of relationships between SLW and CID and the value of CID as an integrative measure of transpiration efficiency (Farquhar et al., 1989), variation in CID relative to leaf thickness may provide insight into resource capture and partition. Additionally, nitrogen isotope discrimination (referred to as D15N hereafter) may potentially reveal how short-term variation in nitrogen cycling, nitrogen metabolism, and responses to water deficit impacts canopy reflectance traits like normalized difference vegetative index (NDVI), a general measure of crop health and biomass (Tucker, 1979; Craine et al., 2015).

The thickness of a leaf is initially established following a phase of rapid thickening growth (Maksymowych, 1973). In addition to water deficits, low temperature and high irradiance are associated with thicker leaves (Van Volkenburgh and Davies, 1977; Rawson et al., 1987; Nobel, 1999; Evans and Poorter, 2001). Although elevated atmospheric CO_2_ is usually expected to increase SLW due to accumulation of assimilate (Poorter and Perez-Soba, 2002), Thomas and Harvey (1983) reported that thicker leaves in soybean (*Glycine max* L. Merr.) under elevated CO_2_ resulted from the formation of an additional layer of palisade mesophyll.

In cotton, leaf thickness increases with main stem node position but plateaus by node 12 or 13 (Gausman et al., 1971). At the species level, variation has been observed in the diploid, A-genome donors of *G. arboreum* L. and *G. herbaceum* L. with older leaves forming an additional layer of palisade mesophyll cells on the abaxial (lower) side (Morey et al., 1974; Bhatt and Andal, 1979; Leidi et al., 1999). With respect to cultivated cotton, Morey et al. (1974) reported differences in leaf thickness among 17 lines representing the perennial races of *G. hirsutum* L. as well as two upland cultivars under greenhouse conditions measured in 2 and 6 month old plants.

A concern related to selection of leaf traits that might affect canopy reflectance properties is that of developmental correlations; traits affecting cell sizes within leaves may also impact the cells sizes of other tissues (White and Gonzalez, 1990; John et al., 2013). Thus, selection for traits related to leaf spectral reflectance might have undesirable effects on other useful plant traits. In perennial ryegrass (*Lolium perenne* L.), divergent selection for mesophyll cell size resulted in heavier seed and greater shoot dry matter for small-cell size selections (Wilson and Cooper, 1970). In cotton, a particular concern is fiber quality. Because cotton fibers are formed from unicellular epidermal hairs (Mauney, 1984), selection affecting leaf thickness also might affect epidermal hairs. Although associations among fiber quality traits and agronomic factors have been examined (Ulloa, 2006; Dabbert et al., 2017) research on how genetic variation in cell size might affect fiber quality appears to be lacking.

Recent research using proximal sensing in cotton demonstrated that spectral reflectance indices measured on crop canopies can identify genetic differences among cotton lines under well-watered and water-limited deficit conditions (Andrade-Sanchez et al., 2014; Pauli et al., 2016a). However, there exists knowledge gaps in understanding how the physical and biochemical properties of the cotton canopy itself impact canopy reflectance detected using HTP approaches to characterize genetically diverse germplasm under contrasting irrigation regimes across multiple years. The main objectives of the research described herein were to determine (1) whether genetic variation in leaf thickness or related traits affected canopy spectral reflectance measured using HTP methods, (2) whether relations existed between leaf thickness and other crop traits either through physiological or developmental correlations, and (3) identify regions of the cotton genome controlling variation in leaf thickness.

## Materials and methods

All measurements were made on two populations of cotton. The upland (*Gossypium hirsutum* L.) set was the TM-1 × NM24016 mapping population (Percy et al., 2006; Gore et al., 2012) of 95 recombinant inbred lines (RILs). Of the parents used to create this population, TM-1 is the current *G. hirsutum* genetic standard, whose genome was recently sequenced (Zhang et al., 2015), and represents the upland ideotype in terms of relative vigor, high fertility, uniformity, and fruiting habit (Kohel et al., 1970). NM24016, in contrast, is an inbred line derived from an interspecific cross between *G. hirsutum* and *G. barbadense* with approximately 37% genomic similarity, based on DNA marker analysis, to *G. barbadense*. Morphologically, its traits display an intermediate phenotype between the two species (Cantrell and Davis, 2000). The second population was a diversity panel comprised of 25 Pima (*Gossypium barbadense* L.) lines released from 1918 to 2009, capturing a wide range of phenotypic diversity from Arizona with two additional lines originating from the Caribbean Islands. The two populations were grown in three sets of field trials from 2010 to 2012 at Maricopa, AZ (lat. 33.070° N, long. 111.974° W, elev. 360 m) on a Casa Grande sandy loam (fine-loamy, mixed, superactive, hyperthermic Typic Natrargids). Experimental designs, crop management and phenotyping were described previously (Carmo-Silva et al., 2012; Andrade-Sanchez et al., 2014; Thorp et al., 2015; Pauli et al., 2016a). Briefly, well-watered (WW) and water-limited (WL) irrigation trials of the upland lines were arranged as 11 × 10 (0.1) α-lattices with two replicates. Pima lines were arranged as 5 × 5 (0.1) α-lattices with four replicates. To reduce border effects, a commercial upland or Pima cultivar was planted on the sides of each replicate. One-row plots were 8.8 m long and 1 m wide with a 0.61 m alley at row ends. Plant density for both populations was ~4.1 plants m^−2^ after thinning.

Crop management followed recommended practices for the desert southwest. Crops were furrow irrigated for germination and seedling establishment, and subsequently irrigated via subsurface drip. Irrigations for the well-watered (WW) regime were scheduled to refill the depleted soil water of the cotton root zone based on calculated crop evapotranspiration using the dual crop coefficient procedures of the Food and Agriculture Organization Paper 56 (Allen et al., 1998). Allowable depletion of the total available root zone soil water was set at 35% active rooting zone, with a few final adjustments to the soil water balance made based on actual soil moisture as measured via neutron probe readings. Weekly soil moisture content readings were made from 0.1 to 1.5 m, in 0.2-m increments. When 50% of plots had reached first flower, the water-limited (WL) irrigation regime was imposed by providing 50% of the water applied to the WW regime.

**Figure 1:**
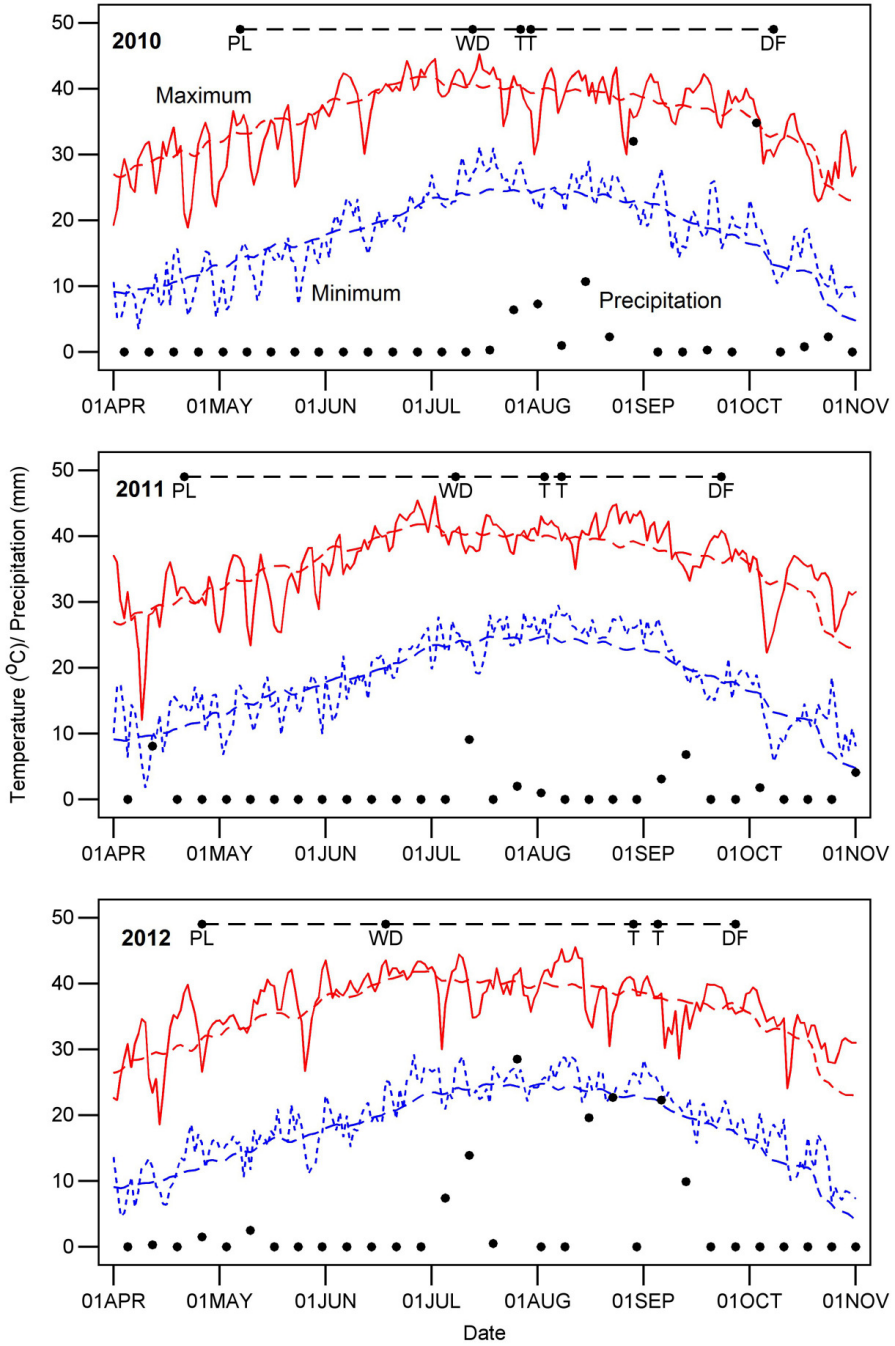
Daily weather during the 3 years of cotton experiments. Letters along the dashed line at the top of the graph for each year indicate the time from planting (PL) to chemical defoliation (DF), the date that the water-limited irrigation regime was initiated (WD) and the start and end dates for measurements of leaf thickness (T). The red and blue colored lines represent the maximum and minimum air temperature, respectively. Black dots denote the precipitation amounts and days on which it occurred.

Dates for crop management and measurements are summarized in Supplementary Table 1, and key dates are indicated for each year on Figure 1, which also shows temperature and precipitation for each year. Samples for leaf thickness and SLW were acquired before 10:30 AM Mountain Standard Time (MST) to avoid possible changes in thickness related to progressive water loss during the day. Leaf thickness (THK, reported as mm) was measured on five to eight fully-expanded leaves per plot from the uppermost part of the canopy, sampling at the third or fourth interveinal region from the leaf apex. Measurements were made using a hand-held micrometer (Mitutoyo Digital Micrometer Model 293-185, Kawasaki, Japan) with a digital display and a clutch that ensured uniform pressure. Plot positions and micrometer readings were dictated in the field using Philips Voice Tracer 667/00 (Koninklijke Philips N.V., Amsterdam) digital recorders, and the resulting audio was converted to digital text via the speech recognition software Dragon Naturally Speaking (version 11 Premium; Nuance Communications, Inc., Burlington, MA, USA). We estimated a reference thickness as the mean of BLUEs for the WW regimes across the 3 years because our underlying hypothesis is that leaf thickness is a constitutive trait that affects other traits.

Relative chlorophyll content (SPAD, unitless) was obtained for five to eight leaves per plot, sampled as for thickness, using a Minolta SPAD Meter 502 (Konica Minolta Sensing, Inc., Japan). Additionally, actual chlorophyll *a* (Chl_a) and *ab* (Chl_ab) concentrations were measured using a protocol adopted from Porra et al. (1989). Harvested leaf disks, two samples per plant, were frozen to −80°C until time of processing at which point 1 mL of 100% methanol was added to sample tubes and mixed well. Samples were then incubated at 4°C for approximately 48 h and then mixed and spun down so that 200 μL of supernatant could be transferred to a microtiter plate and absorbance read at 652 and 665 nm using a Bio-Tek Microplate reader (Bio-Tek, Winooski, VT). Concentrations were reported as μg cm^−2^.

In 2010 only, specific leaf weights were estimated for five, 1-cm diameter leaf disks cut with a leaf punch that deposited samples into a glass vial, again sampled from fully-expanded leaves in the upper canopy. The vials were refrigerated while transported to the laboratory for fresh weight determination. The weighed samples were then oven dried (70°C) and re-weighed for calculation of specific leaf weight. Estimates of specific leaf weight were reported on fresh (SLW_fr_) and dry (SLW_dr_) bases in units of g m^−2^.

To measure CID, leaf tissue samples were taken from six representative plants within each plot with samples taken from the upper lobe of a fully expanded leaf near the fourth node of the plant. Leaf discs were taken with a 6 mm punch and sampled directly into 1.2 mL tubes of a 96-well plate which were then promptly stored on ice in a Styrofoam cooler until brought out of the field; tissue samples were then properly preserved for subsequent analyses. Carbon isotope composition analysis was performed by the University of California, Davis Stable Isotope Facility (Davis, CA, US). In 2010, leaf discs were collected on day 231 (Julian calendar), which corresponded with the end of cotton boll development and fill. In 2011 and 2012, leaf discs were collected on days 251 and 249 (Julian calendar), respectively, which coincided with cotton fiber development and elongation. Dried leaf discs were ground to a fine powder followed by weighing and placing 1–2 mg of subsamples into foil capsules. Carbon isotope composition was determined with an isotope ratio mass spectrometer (Sercon Ltd., Cheshire, UK) and calculated as δ^13^C (‰) relative to the international Vienna Pee Dee Belemnite (V-PDB) reference standard (Farquhar et al. (1989). Carbon isotope discrimination (CID, reported as part per thousand, mole fraction, ‰) was then calculated by the method of Farquhar et al. (1989) using the following equation:

(1) $$\text{CID}=[(\delta_{\text{a}}-\delta_{\text{p}})]/[1+(\delta_{\text{p}}/1,000)]$$

where δ_a_ and δ_p_ represent the stable carbon isotope composition of the atmosphere and the plant tissue sample, respectively. On the V-PDB scale, a value of −8‰ was used for the free atmospheric CO_2_ concentration, δ_a_. For nitrogen discrimination (D15N, ‰), values were calculated relative to atmospheric composition.

At the end of each growing season prior to mechanical harvesting, 25 bolls were sampled from each plot and processed using a laboratory 10-saw gin to collect fiber for analysis of quality. Fiber quality measurements for upland cotton were made using an Uster HVI 1000 (High Volume Instrument, Uster, Charlotte, NC) at Cotton Incorporated (Cary, NC). Fiber quality measurements for the Pima population were also made on an Uster HVI 1000 but conducted at the Fiber and Biopolymer Research Institute at Texas Tech University (Lubbock, TX). The traits measured were fiber elongation (ELO, percent), strength (STR, kN m kg^−1^), uniformity (percent), micronaire (unit), and length (upper half mean, UHM, mm). However, in the current work, fiber strength, elongation, and upper half mean are discussed, as these traits are more representative of the underlying biological process of carbon fixation.

A field-based, high-throughput phenotyping (HTP) system was used to rapidly collect proximally sensed canopy data to evaluate numerous canopy phenotypes over the 2010–12 growing seasons. The design, development, operational parameters, and field evaluation of this phenotyping platform have been previously described in detail in Andrade-Sanchez et al. (2014) and Pauli et al. (2016a). Briefly, a LeeAgra AvengerPro modified high-clearance small plot spray rig with a front, horizontal boom was used to move identical sets of sensors over four adjacent rows, with geographic positions measured with an RTK-GPS returning cm-level accuracy (~2 cm resolution). Each set of sensors included ultrasonic proximity sensors to measure canopy height, infrared radiometers to measure canopy temperature, and active light multi-spectral crop canopy sensors to measure canopy reflectance. For the present study, only the data collected by the multi-spectral crop canopy sensor (Crop Circle ACS 470, Holland Scientific, Lincoln, NE, US) were used, which provided canopy reflectance (ρ) in three 10 nm wavebands with band centers at 670, 720, and 820 nm. The wavelength data collected from the CropCircle multi-spectral sensors were used to calculate normalized difference vegetation index (NDVI, unitless) as follows:

(2) $$\text{NDVI}=(\rho_{NIR}-\rho_{red})/(\rho_{NIR}+\rho_{red})$$

where ρ_*NIR*_ is the spectral reflectance at wavelength 820 nm in the near-infrared waveband region and ρ_*red*_ is the spectral reflectance at wavelength 670 nm in the red waveband region. Measurements were taken in the early morning (0700), midmorning (1000 or 1100), afternoon (1300), and/or late afternoon (1500) with all times reported in MST. The time of day (0700, 1000, 1100, 1300, or 1500) that data were collected is referred as time of day (TOD), while the actual time, measured in minutes, that a measurement was taken is referred to as time of measurement (TOM). Only the data collected nearest to the time of leaf thickness measurements are reported; the HTP system required ~0.75 h to traverse the complete set of experimental plots.

## Statistical analyses

Best linear unbiased estimators (BLUEs) were estimated for each trait via iterative mixed linear model fitting using ASReml-R version 3.0 (Gilmour et al., 2009), as detailed in Pauli et al. (2016a). To assess whether the leaf thickness, physiological traits, fiber quality, and post-processed NDVI data contained outliers, we initially fitted a simplified mixed linear model for each trait using the MIXED procedure in SAS for Windows version 9.4 (SAS Institute, Cary, NC). For the physiological and fiber quality traits, the fitted model for an individual trait included the main effects of genotype and irrigation regime with their two-way interaction as fixed effects; year, year-by-genotype interaction, replication nested within irrigation regime, column nested within the two-way interaction of replication and irrigation regime, and block nested within the two-way interaction of replication and irrigation regime were included as random effects. The fitted model used for NDVI outlier removal included the main effects of genotype and irrigation regime with their two-way interaction as fixed effects; replication nested within irrigation regime and block nested within the two-way interaction of replication and irrigation regime were included as random effects. For both models, degrees of freedom were calculated via the Satterthwaite approximation. The Studentized deleted residuals (Neter et al., 1996) obtained from these mixed linear models were examined to detect outliers and remove them for subsequent analyses. For the NDVI data sets, plot-level averages were calculated with the MEANS procedure in SAS for Windows version 9.4 (SAS Institute, Cary, NC).

For each physiological and fiber quality trait, an iterative mixed linear model fitting procedure was conducted across years in ASReml-R version 3.0 (Gilmour et al., 2009):

(3) $$\begin{array}{l} {\text{Y}}_{ijklmn}=\mu +{\text{year}}_{i}+{\text{irg}}_{j}+{\left(\text{irg}\times \text{year}\right)}_{\text{ij}}+\text{rep}{\left(\text{irg}\times \text{year}\right)}_{ijk}\\ \;+\text{ column}{\left(\text{rep}\times \text{irg}\times \text{year}\right)}_{ijkl}\\ \;+\text{ block}{\left(\text{rep}\times \text{irg}\times \text{year}\right)}_{\text{ijkm}}\\ \;+\;{\text{genotype}}_{n}+{\left(\text{genotype}\times \text{year}\right)}_{in}\\ \;+\;{\left(\text{genotype}\times \text{irg}\right)}_{jn}\\ \;+\;(\text{genotype}\times \text{irg}\times \text{year}{)}_{ijn}+\varepsilon_{ijklmn} \end{array}$$

in which Y_*ijklmn*_ is an individual phenotypic observation; μ is the grand mean; year_*i*_ is the effect of the *i*th year; irg_*j*_ is the effect of the *j*th irrigation regime (WW or WL); (irg × year)_*ij*_ is the interaction effect between the *i*th year and *j*th irrigation regime; rep(irg × year)_*ijk*_ is the effect of the *k*th replication within the *j*th irrigation regime within the *i*th year; column(rep × irg × year)_*ijkl*_ is the effect of the *l*th plot grid column within the *k*th replication within the *j*th irrigation regime within the *i*th year; block(rep × irg × year)_*ijkm*_ is the effect of the *m*th incomplete block within the *k*th replication within the *j*th irrigation regime within the *i*th year; genotype_*n*_ is the effect of the *n*th genotype; (genotype × year)_*in*_ is the interaction effect between the *n*th genotype and the *i*th year; (genotype × irg)_*jn*_ is the interaction effect between the *n*th genotype and the *j*th irrigation regime; (genotype × irg × year)_*ijn*_ is the effect of the three way interaction effect between *n*th genotype, the *j*th irrigation regime, and the *i*th year; and ε_*ijklmn*_ is the random error term following a normal distribution with mean 0 and variance σ^2^. The model terms rep(irg × year)_*ijk*_, column(rep × irg × year)_*ijkl*_, and block(rep × irg × year)_*ijkm*_ were fitted as random effects while all other terms were fitted as fixed effects. Likelihood ratio tests were conducted to remove all terms from the model that were not significant at α = 0.05 (Littell et al., 2006).

For NDVI an iterative repeated measures mixed linear model fitting procedure was conducted separately for each day in ASReml-R version 3.0 (Gilmour et al., 2009):

(4) $$\begin{array}{l} {\text{Y}}_{ijklmo}=\mu +{\text{tod}}_{i}\text{+ }{\text{irg}}_{j}\text{+ }{\left(\text{tod}\times \text{irg}\right)}_{ij}\\ \;+\text{ rep}{\left(\text{irg}\times \text{tod}\right)}_{ijk}+\text{ column}{\left(\text{rep}\times \text{irg}\times \text{tod}\right)}_{ijkl}\\ \;+\text{ block}{\left(\text{rep}\times \text{irg}\times \text{tod}\right)}_{ijkm}\\ \;+\text{ tom}{\left(\text{irg}\times \text{tod}\right)}_{ijn}\\ \;+\;{\text{genotype}}_{o}+\;{\left(\text{genotype}\times \text{tod}\right)}_{io}+\\ \;+\;{\left(\text{genotype}\times \text{irg}\right)}_{jo}+\;{\left(\text{genotype}\times \text{irg}\times \text{tod}\right)}_{ijo}\\ \;+\;\varepsilon_{ijklmno,}\\ \text{ with }\varepsilon_{ijklmno}\text{ equal to }Var(\varepsilon_{ijklmno})=\sigma^{2},Cov(\varepsilon_{ijklmno,}\\ \;\varepsilon_{i^{\prime }jklmno})=\rho \sigma^{2},i\neq i^{\prime } \end{array}$$

in which Y_*ijklmo*_ is an individual plot-level average; μ is the grand mean; tod_*i*_ is the effect of the *i*th TOM within a day; irg_*j*_ is the effect of the *j*th irrigation regime (WW or WL); (tod × trt)_*ij*_ is the effect of the interaction between the *i*th TOM within a day and the *j*th irrigation regime; rep(irg × tod)_*ijk*_ is the effect of the *k*th replication within the *j*th irrigation regime within the *i*th TOM within a day; column(rep × irg × tod)_*ijkl*_ is the effect of the *l*th plot grid column within the *k*th replication within the *j*th irrigation regime within the *i*th TOM within a day; block(rep × irg × tod)_*ijkm*_ is the effect of the *m*th incomplete block within the *k*th replication within the *j*th irrigation regime within the *i*th TOM within a day; tom(irg × tod)_*ijn*_ is the effect of the *n*th minute the measurement was taken within the *j*th irrigation regime within the *i*th TOM within a day; genotype_*o*_ is the effect of the *o*th genotype; (genotype × tod)_*io*_ is the effect of the interaction between the *o*th genotype and the *i*th TOM within a day; (genotype × irg)_*jo*_ is the effect of the interaction between the *o*th genotype and the *j*th irrigation regime; (genotype × irg × tod)_*ijo*_ is the effect of the interaction between the *o*th genotype, the *j*th irrigation regime, and the *i*th TOM within a day; and ε_*ijklmno*_ is the random error term following a normal distribution with mean 0 and variance σ^2^. The residual variance, ε_*ijklmno*_, was modeled using a correlated error variance structure that incorporated a constant, non-zero, correlation term (ρ) among error terms to account for correlation among multiple measures on the same experimental unit. The following terms were fitted as fixed effects in the model: tod_*i*_; genotype_*o*_; irg_*j*_; (genotype × irg)_*jo*_; (genotype × tod)_*io*_; (tod × irg)_*ij*_; and (genotype × irg × tod)_*ijo*_. All other terms were fitted as random effects. Likelihood ratio tests were conducted to remove all terms from the model that were not significant at α = 0.05 (Littell et al., 2006).

For each trait, any remaining influential outliers from the final fitted model were detected on the basis of the DFFITS criterion (Neter et al., 1996; Belsley et al., 2004) in ASReml-R version 3.0 (Gilmour et al., 2009). Once influential observations were removed, the final model (2 or 3) for each trait was refitted to estimate a BLUE for each genotype across years (fiber quality and physiological traits) or within a day (NDVI) for the separate irrigation regimes. Sequential tests of fixed effects were conducted with degrees of freedom being calculated with the Kenward and Rogers approximation (Kenward and Roger, 1997) in ASReml-R version 3.0 (Gilmour et al., 2009).

For each trait, broad-sense heritability on an entry-mean basis (*Ĥ*^2^) or repeatability (Piepho and Möhring, 2007) was estimated to provide a measure of how much phenotypic variation among genotypes was due to heritable genetic effects rather than to environmental or measurement error for the Pima population in the absence of pedigree or molecular marker data; in the context of the upland population (biparental family) this is only referred to as broad-sense heritability on an entry-mean basis (*Ĥ*^2^, referred to as heritability hereafter). Heritability was estimated for the separate irrigation regimes using a mixed linear model. To estimate heritability, models (2) and (3) were reformulated to remove the irrigation regime term. Next, all terms were then fitted as random effects in order to obtain variance component estimates. The variance component estimates from each final model for fiber quality and physiological traits were used to estimate *Ĥ*^2^ (Holland et al., 2003) as follows:

(5) $${\hat{H}}^{2}=\frac{\hat{\sigma_{g}^{2}}}{\hat{\sigma_{g}^{2}}+\frac{\hat{\sigma_{gy}^{2}}}{n_{year}}+\frac{\hat{\sigma_{\varepsilon }^{2}}}{n_{plot}}}=\frac{\hat{\sigma_{g}^{2}}}{\hat{\sigma_{p}^{2}}}$$

where $\hat{\sigma_{g}^{2}}$ is the estimated genetic variance, $\hat{\sigma_{gy}^{2}}$ is the estimated variance associated with genotype-by-year variation, $\hat{\sigma_{\varepsilon }^{2}}$ is the residual error variance, *n*_*year*_ is the harmonic mean of the number of years in which each genotype was observed and *n*_*plot*_ is the harmonic mean of the number of plots in which each genotype was observed. The denominator of Equation (5) is equivalent to the phenotypic variance, $\hat{\sigma_{p}^{2}}$. The variance component estimates from the final model for NDVI were used to estimate *Ĥ*^2^ (Holland et al., 2003) as follows:

(6) $${\hat{H}}^{2}=\frac{\hat{\sigma_{g}^{2}}}{\hat{\sigma_{g}^{2}}+\frac{\hat{\sigma_{\varepsilon }^{2}}}{n_{plot}}}=\frac{\hat{\sigma_{g}^{2}}}{\hat{\sigma_{p}^{2}}}$$

where all terms are as previously defined above.

Because the objectives of this study focused on understanding how genotypic differences in leaf thickness impact other phenotypes, we calculated a reference leaf thickness (reference thickness) that represented the expected phenotype under ideal conditions, i.e., no water deficit. To accomplish this, an overall BLUE was calculated for each genotype using the measurements from the WW regime. This was expected to mitigate the effects of water deficit on leaf thickness thereby minimizing confounding environmental factors that could adversely bias the estimate.

To investigate the genetic relationship among the traits, we estimated the genotypic correlations (${\hat{r}}_{gij}$) and their standard errors in the RIL population with respect to the two irrigation regimes. Due to the uncontrolled, multiple levels of relatedness between lines, this analysis was not possible to conduct with the Pima population. To carry out the analysis, we used a multivariate restricted maximum likelihood (REML) estimation procedure implemented in PROC MIXED of SAS version 9.4 (SAS Institute., Cary, NC) as described by Holland (2006). Prior to model fitting, the BLUEs calculated for the individual years within irrigation regime were standardized to have a mean of zero and a standard deviation of one; this was done using PROC STANDARDIZE in SAS to assist in model convergence. The model used for the RIL population to estimate variance components was as follows:

(7) $$\begin{array}{l} {\text{Y}}_{ijkl}=\mu +\text{year}{\left(\text{trait}\right)}_{ijk}+{\text{genotype}}_{l}+\\ \;{\left(\text{year}\times \text{genotype}\right)}_{kl}+\varepsilon_{ijkl} \end{array}$$

where Y_*ijkl*_ are the paired BLUEs for the *i*th and *j*th traits in the *k*th year for the *l*th genotype; μ is the multivariate grand mean; year(trait)_*ijk*_ is the effect of the *k*th year on the combined *i*th and *j*th traits; genotype_*l*_ is the effect of the *l*th genotype; (year × genotype)_*kl*_ is the effect of the interaction between the *k*th year and the *l*th genotype; and ε_*ijkl*_ is the random error term. The random effect terms in the model were genotype_*l*_ and (year × genotype)_*kl*_ while the only fixed effect was year(trait)_*ijk*_. To estimate the covariance associated with the paired *i*th and *j*th traits for the estimated BLUEs per each genotype, the REPEATED statement was used.

The estimated variance components form Equation (7) were used in the following formula to derive the genotypic correlations (${\hat{r}}_{gij}$):

(8) $${\hat{r}}_{gij}=\frac{{\hat{\sigma }}_{Gij}}{{\hat{\sigma }}_{Gi}{\hat{\sigma }}_{Gj}}$$

where ${\hat{\sigma }}_{Gij}$ is the estimated genotypic covariance between traits *i* and *j*, ${\hat{\sigma }}_{Gi}$ is the estimated genotypic standard deviation of trait *i* and ${\hat{\sigma }}_{Gj}$ is the estimated genotypic standard deviation of trait *j*.

To explore the effect of reference leaf thickness on specific traits, once effects of year and irrigation regime were accounted for, linear regression was performed using the GLM procedure of SAS with the model:

(9) $${\text{Y}}_{ijk}=\mu +\text{irg}{\left(\text{year}\right)}_{ij}+{\text{thickness}}_{k}+\varepsilon_{ijk}$$

where Y_*ijk*_ is the BLUE for a given trait (as opposed to value for individual replicates), irg(year)_*ij*_ is the effect of the *j*th year nested within the effect of the *i*th irrigation regime, thickness_*k*_ is the reference thickness for the *k*th genotype, and ε_*ijk*_ is the random error term following a normal distribution with mean 0 and variance σ^2^. Sums of squares are sequential (Type I) to indicate the effect of variation in leaf thickness once expected large effects of irrigation regime nested within year are considered.

Within an irrigation regime, the Pearson's correlation coefficients (*r*) were estimated using PROC CORR in SAS version 9.4 (SAS Institute Inc., Cary, NC) to examine relations between sets of BLUEs for different traits.

To identify the regions of the cotton tetraploid genome controlling phenotypic variation in leaf thickness, we performed quantitative trait loci (QTL) mapping within the upland RIL population. Due to lack of genotypic data and appropriate population construction/composition, QTL mapping within the Pima population was not possible. The genotyping and linkage map construction for the TM-1 × NM24016 RIL population has been previously described in detail in Gore et al. (2014). Briefly, the linkage map consisted 841 molecular markers assigned to 117 linkage groups covering approximately 50% of the cotton genome; this generated a linkage map ~2,061 cM in length.

The BLUEs for leaf thickness were used individually to map additive QTL effects with respect to the WL and WW irrigation regimes using inclusive composite interval mapping (ICIM) (Li et al., 2007, 2015) for biparental populations implemented in the software IciMapping v 4.0 (https:www.integratedbreeding.net). To determine the logarithm of odds (LOD) threshold value for declaring significance, a permutation procedure was run 1,000 times (Churchill and Doerge, 1994) within the IciMapping software to achieve an experiment-wise Type I error rate of α = 0.05.

## Results

**Figure 2:**
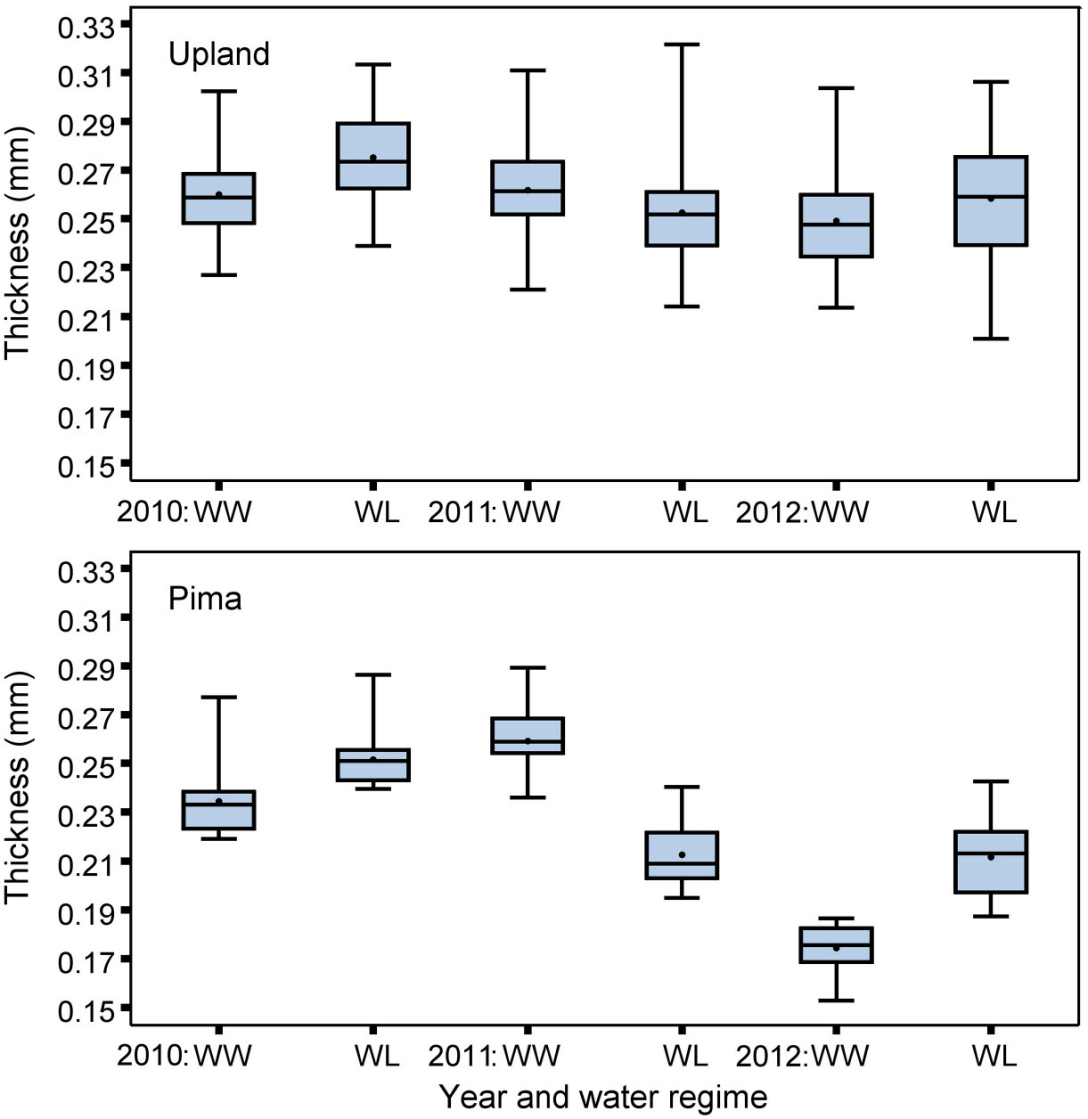
Boxplots of BLUEs for leaf thickness measured with micrometer for the upland recombinant inbred lines and Pima lines, considering well-watered (WW) and water-limited (WL) irrigation regimes in 2010, 2011, and 2012.

The upland and Pima cotton lines showed large variation in leaf thickness (Table 1, Figure 2). Comparing the two sets of germplasm, the upland lines had thicker leaves (three year averages of 0.26 and 0.26 mm for the WL and WW regimes, respectively) than the Pima lines (0.23 and 0.22 mm for the WL and WW regimes, respectively). No mean effect of the irrigation regime on thickness was found for either population (*P* > 0.05, Table 2), but genotype-by-irrigation regime effects were detected for both populations (*P* < 0.01 for the upland and *P* < 0.0001 for the Pima). For both dry and fresh SLW, a trait that generally tracks well with leaf thickness, the irrigation regime effect was highly significant (*P* < 0.001) for the Pima population but nonsignificant (*P* > 0.05) for the upland population. The effect of the individual years on thickness was large for Pima (*P* < 0.001), whereas for the upland population, no year effect was detected (*P* > 0.05), but again, large genotype-by-year effects were found for both populations (Table 2). The broad-sense heritability of leaf thickness was generally high (>0.60) across the years and irrigation regimes.

**Table 1:** Mean, minimum, maximum, and standard deviation of best linear unbiased estimators (BLUEs) for traits evaluated for the upland recombinant inbred line (RIL) and Pima populations tested under two irrigation regimes, water-limited (WL) and well-watered (WW) conditions.

**Trait**	**Year**	**Irrigation regime**	**Upland**						**Pima**					
			**Mean**	**Min**	**Max**	**SD**	***Ĥ^2^***	**SE of *Ĥ^2^***	**Mean**	**Min**	**Max**	**SD**	***Ĥ^2^***	**SE of *Ĥ^2^***
THK (mm)	2010	WW	0.26	0.23	0.30	0.01	0.67	0.05	0.23	0.22	0.28	0.01	0.88	0.04
		WL	0.27	0.24	0.31	0.02	0.76	0.04	0.25	0.24	0.29	0.01	0.94	0.02
	2011	WW	0.26	0.22	0.31	0.02	0.37	0.10	0.26	0.24	0.29	0.01	0.26	0.25
		WL	0.25	0.21	0.32	0.02	0.82	0.03	0.21	0.20	0.24	0.01	0.65	0.12
	2012	WW	0.25	0.21	0.30	0.02	0.73	0.04	0.17	0.15	0.19	0.01	0.67	0.12
		WL	0.26	0.20	0.31	0.02	0.72	0.05	0.21	0.19	0.24	0.02	0.84	0.05
SLW_fr_ (g m^−2^)	2010	WW	236.22	195.66	282.32	19.09	0.00	0.00	183.31	169.95	215.44	10.85	0.39	0.17
		WL	238.00	182.77	317.27	25.51	0.42	0.12	203.65	187.91	240.67	11.78	0.50	0.15
SLW_dr_ (g m^−2^)	2010	WW	49.57	42.08	58.63	3.57	0.12	0.16	45.52	41.77	49.02	1.84	0.35	0.16
		WL	55.17	43.64	73.39	5.22	0.39	0.14	48.88	44.42	52.63	1.90	0.28	0.18
Chl_a (ug cm^−2^)	2010	WW	32.88	26.51	39.75	2.48	0.58	0.08	34.92	30.13	38.60	2.04	0.68	0.12
		WL	39.08	31.58	52.27	3.39	0.41	0.11	39.95	36.28	43.40	2.16	0.66	0.12
	2011	WW	30.39	23.63	37.50	2.98	0.22	0.12	31.84	26.95	37.20	2.10	0.74	0.08
		WL	29.67	23.90	35.98	2.49	0.17	0.14	33.13	28.48	38.70	2.25	0.64	0.12
	2012	WW	30.32	25.26	38.28	2.52	0.53	0.08	30.67	28.00	36.40	2.03	0.42	0.19
		WL	32.22	27.13	39.87	2.38	0.32	0.14	30.76	26.36	34.09	1.98	0.24	0.26
Chl_ab (ug cm^−2^)	2010	WW	40.51	33.18	49.30	3.01	0.58	0.08	44.34	37.90	49.15	2.58	0.70	0.11
		WL	48.17	39.02	64.28	4.16	0.43	0.11	50.78	46.25	55.68	2.81	0.69	0.11
	2011	WW	37.54	29.66	45.63	3.57	0.17	0.13	40.03	33.71	46.31	2.55	0.75	0.08
		WL	36.67	29.50	46.08	3.16	0.23	0.13	41.98	35.91	48.88	2.86	0.63	0.12
	2012	WW	36.88	30.75	46.84	2.98	0.53	0.08	38.66	35.30	45.25	2.48	0.43	0.18
		WL	39.77	33.48	49.24	2.95	0.40	0.10	38.87	33.95	42.89	2.37	0.22	0.26
SPAD (unitless)	2010	WW	38.36	33.09	43.21	2.02	0.68	0.05	35.38	32.13	39.96	1.54	0.84	0.05
		WL	40.20	35.61	45.24	1.93	0.71	0.04	37.70	35.49	41.33	1.31	0.84	0.05
	2011	WW	36.15	29.35	45.93	2.71	0.67	0.05	30.91	27.77	35.37	1.99	0.76	0.08
		WL	39.72	33.03	45.92	2.51	0.70	0.05	33.26	30.57	37.57	1.76	0.85	0.05
	2012	WW	35.62	29.89	41.93	2.71	0.79	0.03	31.56	28.12	34.79	1.90	0.86	0.04
		WL	37.92	31.25	44.77	2.46	0.71	0.04	33.54	29.51	37.04	2.04	0.86	0.04
CID (‰)	2010	WW	20.47	19.59	21.33	0.35	0.74	0.05	21.15	19.66	21.54	0.39	0.90	0.04
		WL	20.65	19.50	21.36	0.39	0.73	0.05	20.59	19.60	21.20	0.39	0.87	0.05
	2011	WW	20.21	18.79	21.12	0.41	0.65	0.07	20.67	18.78	21.77	0.58	0.92	0.03
		WL	20.01	18.88	21.06	0.36	0.48	0.11	20.21	18.64	20.99	0.51	0.86	0.05
	2012	WW	20.79	19.34	21.86	0.47	0.76	0.06	21.49	19.87	22.22	0.48	0.90	0.04
		WL	20.10	18.96	21.04	0.42	0.76	0.05	20.35	18.54	21.31	0.52	0.88	0.05
D15N (‰)	2010	WW	3.57	2.98	4.23	0.27	0.38	0.13	–	–	–	–	–	–
		WL	2.93	1.75	3.52	0.36	0.27	0.15	–	–	–	–	–	–
	2011	WW	2.89	2.14	3.92	0.33	0.54	0.10	2.29	1.77	3.13	0.36	0.73	0.10
		WL	2.61	1.69	4.10	0.40	0.67	0.07	1.85	1.49	2.24	0.22	0.50	0.20
	2012	WW	3.00	2.42	3.79	0.29	0.18	0.16	2.84	2.37	3.27	0.24	0.52	0.19
		WL	3.15	2.49	3.97	0.29	0.08	0.16	2.69	2.25	3.13	0.22	0.08	0.40

*Estimates of broad-sense heritability (Ĥ^2^) are on an entry mean basis. Field trials were conducted in 2010–2012 at the Maricopa Agricultural Center located in Maricopa, AZ.SE, standard error*.

**Table 2:** *F*-values and their associated significance values for selected fixed effects from an analysis of variance (ANOVA) for both the upland recombinant inbred line (RIL) and Pima populations for trait data collected from 2010 to 2012 at the Maricopa Agricultural Center.

**Trait**	**Genotype**	**Irrigation regime**	**Year**	**Genotype × Irrigation regime**	**Genotype × Year**
**UPLAND**					
THK	8.22^***^	0.07 ^NS^	0.31 ^NS^	1.45^**^	2.58^***^
SLW_fr_	1.46^*^	0.15 ^NS^	–	1.15 ^NS^	–
SLW_dr_	1.67^**^	1.82 ^NS^	–	1.19 ^NS^	–
Chl_a	3.98^***^	4.79 ^NS^	2.66 ^NS^	1.06 ^NS^	0.89^*^
Chl_ab	4.07^***^	5.78 ^NS^	3.09 ^NS^	1.17 ^NS^	0.88^*^
SPAD	12.20^***^	2.88 ^NS^	0.95 ^NS^	1.34^*^	1.78^***^
CID	9.11^***^	4.78 ^NS^	6.16^*^	1.80^***^	2.43^***^
D15N	3.34^***^	1.51 ^NS^	1.83 ^NS^	0.90 ^NS^	1.55^***^
UHM	67.14^***^	26.12^***^	150.30^***^	1.13 ^NS^	2.53^***^
STR	52.34^***^	4.39^*^	133.40^***^	1.13 ^NS^	2.01^***^
ELO	124.60^***^	0.80 ^NS^	32.36^***^	1.27^*^	2.65^***^
**PIMA**					
THK	6.72^***^	0.00 ^NS^	35.72^***^	2.56 ^***^	2.93^***^
SLW_fr_	3.84^***^	19.43^***^	–	1.11 ^NS^	–
SLW_dr_	1.72^*^	46.15^***^	–	1.08 ^NS^	–
Chl_a	5.85^***^	34.14^***^	3.92 ^NS^	0.63 ^NS^	0.97 ^NS^
Chl_ab	6.09^***^	43.47^***^	3.71 ^NS^	0.65 ^NS^	1.00 ^NS^
SPAD	19.70^***^	17.90^***^	20.49^***^	1.66 ^*^	1.92^***^
CID	28.60^***^	197.00^***^	36.00^***^	1.57^*^	2.17^***^
D15N	1.52 ^NS^	2.44 ^NS^	14.14^**^	1.02 ^NS^	3.63^***^
UHM	76.18^***^	57.40^***^	161.00^***^	1.56^*^	4.79^***^
STR	89.50^***^	2.11 ^NS^	20.33^***^	1.16 ^NS^	1.58^*^
ELO	65.99^***^	11.30^**^	764.30^***^	0.74 ^NS^	3.00^***^

NS *Not Significant at the < 0.05 level*.

* *Significant at the < 0.05 level*.

** *Significant at the < 0.01 level*.

*** *Significant at the < 0.001 level*.

Other leaf physiological traits (chlorophyll *a* and *ab*, SPAD, CID, and D15N) displayed a marked contrast between the upland and Pima populations with respect to the effect of irrigation regime. For chlorophyll content (*a* and *ab*), CID, and SPAD readings, the effect of irrigation regime was nonsignificant for upland but highly significant (*P* < 0.0001; Table 2) for the Pima population. D15N did not vary with irrigation regime and showed no genotype-by-irrigation regime effect for either population. Of these physiological traits, SPAD, CID, and D15N all displayed highly significant (*P* < 0.0001, Table 2) genotype-by-year interaction effects for both populations.

The use of a novel HTP system enabled us to collect NDVI data under actual field conditions on both the upland and Pima populations at multiple times per day over the growing season. In comparing the two populations, the mean NDVI values were not significantly different (two-sided *t*-test, *P* > 0.05, Table 3), and both populations displayed higher values under WW conditions, as expected. Interestingly, in 2010 the Pima population had a larger range of NDVI values but in years 2011 and 2012, the upland population exhibited a much larger range of values; in 2012 alone the range of values was more than twice that of the Pima population. The high estimates of broad-sense heritability (0.80–0.99) demonstrate that NDVI measurements collected by the HTP system were repeatable.

**Table 3:** Mean, minimum, maximum of best linear unbiased estimators (BLUEs) of normalized difference vegetation index (NDVI) for the upland recombinant inbred line (RIL) and Pima populations tested under two irrigation regimes, water-limited (WL) and well-watered (WW) conditions.

**Year**	**DOY^a^**	**TOD^b^**	**Irrigation regime**	**RIL**				**Pima**			
				**Mean**	**Min**	**Max**	**(*Ĥ^2^*)**	**Mean**	**Min**	**Max**	**(*Ĥ^2^*)**
2010	217	0700	WL	0.70	0.39	0.81	0.92	0.69	0.26	0.77	0.99
			WW	0.78	0.69	0.84	0.80	0.77	0.41	0.81	0.94
		1300	WL	0.67	0.31	0.79	0.92	0.60	0.21	0.71	0.99
			WW	0.78	0.68	0.85	0.80	0.76	0.35	0.81	0.94
2011	216	1100	WL	0.63	0.43	0.77	0.91	0.63	0.56	0.79	0.96
			WW	0.67	0.46	0.81	0.82	0.68	0.55	0.80	0.81
		1500	WL	0.65	0.42	0.78	0.91	0.64	0.57	0.80	0.96
			WW	0.68	0.45	0.82	0.82	0.69	0.56	0.81	0.81
2012	243	0700	WL	0.74	0.60	0.84	0.98	0.83	0.79	0.88	0.97
			WW	0.80	0.66	0.85	0.91	0.85	0.83	0.92	0.91
		1000	WL	0.73	0.59	0.84	0.98	0.82	0.76	0.86	0.97
			WW	0.80	0.65	0.86	0.91	0.84	0.81	0.91	0.91
		1300	WL	0.74	0.59	0.85	0.98	–	–	–	–
			WW	0.81	0.66	0.86	0.91	–	–	–	–
		1500	WL	0.74	0.60	0.84	0.98	0.80	0.72	0.85	0.97
			WW	0.81	0.66	0.86	0.91	0.84	0.81	0.90	0.91

*Estimates of broad-sense heritability (Ĥ^2^) are on an entry mean basis. Field trials were conducted in 2010–2012 at the Maricopa Agricultural Center located in Maricopa, AZ*.

a *DOY, day of year (Julian calendar)*.

b *TOD, time of day (Mountain Standard Time, 24 h clock)*.

The three cotton fiber quality traits investigated in this study varied in response to genotype and irrigation regime, with effects ranging from nonsignificant to highly significant (*P* < 0.0001), but year and genotype-by-year effects were all highly significant (*P* < 0.001, Table 2). The heritability values for these three traits were also high with the lowest reported value being 0.81 for fiber elongation in the WW irrigation regime in 2011 (Supplementary Table 2). This finding is not surprising as fiber quality traits are generally highly heritable and exhibit low environmental variance (Pauli et al., 2016a; Dabbert et al., 2017).

**Figure 3:**
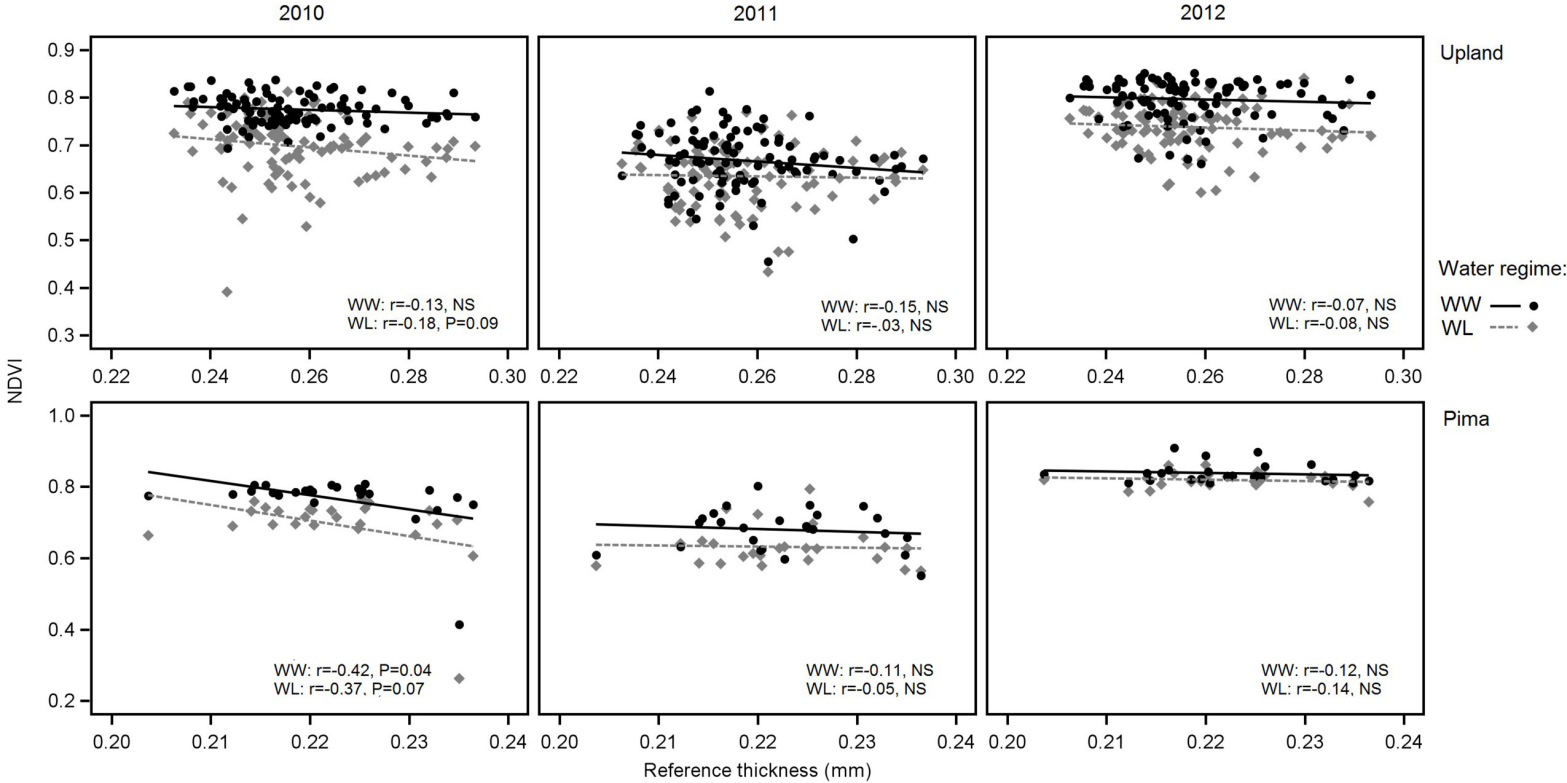
Variation in NDVI in relation to reference leaf thickness for 2010, 2011, and 2012 and the two irrigation regimes. The upper three graphs are for upland RILs, and the lower three are for the Pima diversity panel. Lines indicate regression trends for each irrigation regime. Note difference in scales for upland vs. Pima graphs.

**Figure 4:**
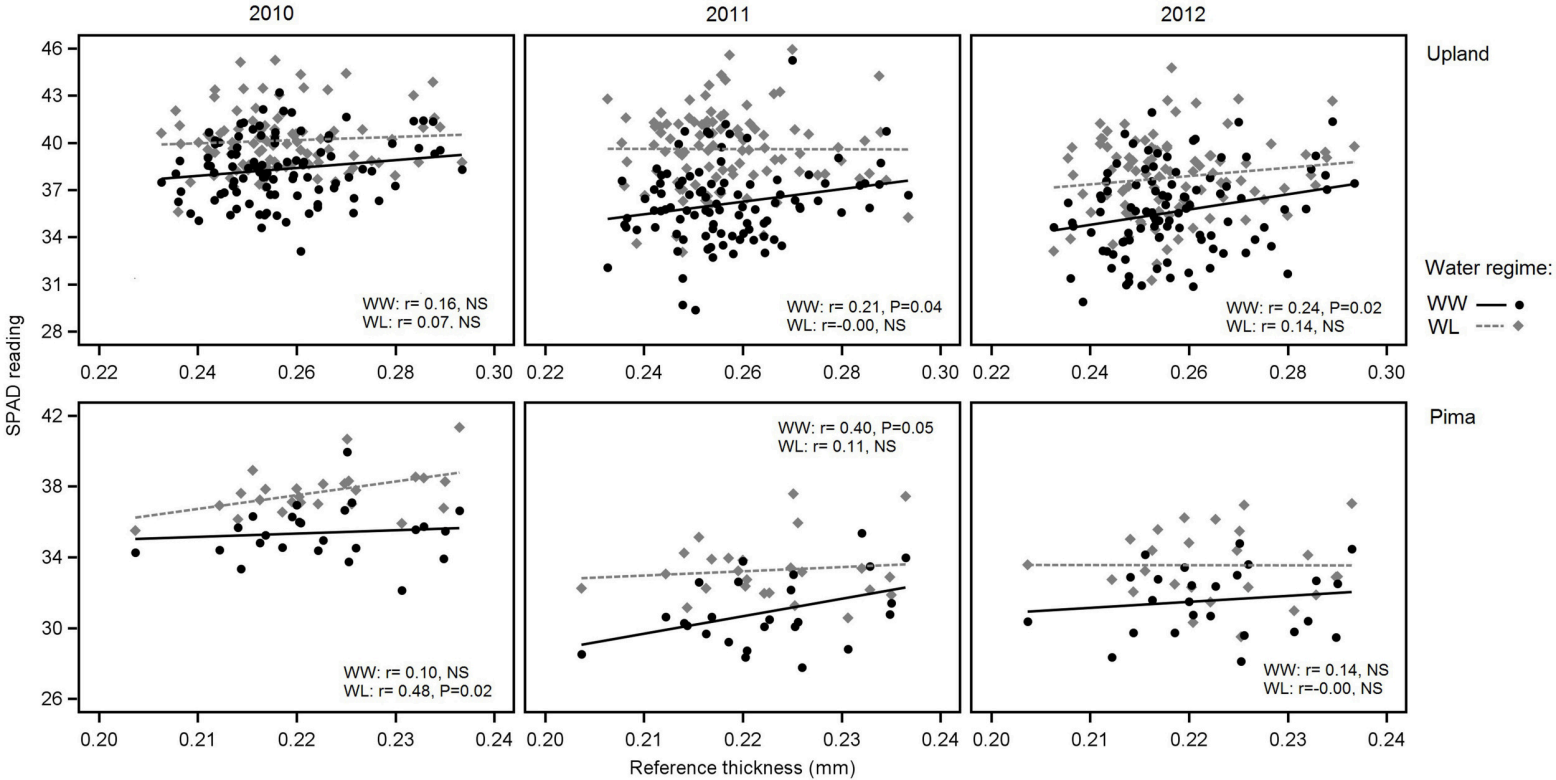
Variation in SPAD readings in relation to reference leaf thickness for 2010, 2011 and 2012 and the two irrigation regimes. The upper three graphs are for upland RILs, and the lower three are for the Pima diversity panel. Lines indicate regression trends for each irrigation regime. Note difference in scales for upland vs. Pima graphs.

In examining relations between reference leaf thickness and individual traits, patterns varied between the two sets of germplasm and in some instances, with year or irrigation regime (Table 4). The two populations also varied for relationships between leaf thickness and NDVI. For NDVI of the Pima population (Figure 3; Table 4), there were highly significant, strong correlations (maximum of −0.73, *P* < 0.001) with leaf thickness but in the upland population, none of the correlations were significant. The correlations between the concentrations of chlorophyll *a* and *ab* with leaf thickness and reference thickness were generally positive in both populations; however, there were more than three times as many significant associations among reference thickness and chlorophyll content (Table 4). The SPAD values also exhibited a positive relationship with leaf thickness, but fewer correlations were significant (Table 4; Figure 4). Specific leaf weight, measured only in 2010, showed varied relations with actual and reference thickness (Supplementary Table 3). Correlations were strongest for SLW_fr_ under WL conditions, and only two of eight correlations were significant for SLW_dr_. As reported for common bean (White and Montes, 2005), associations between SLW and thickness were weaker than implied by studies that assert a direct equivalence between the two traits, thus emphasizing that SLW is an imperfect proxy for leaf thickness.

**Table 4:** Phenotypic correlations (Pearson's) estimated among various leaf and physiological traits for the upland recombinant inbred line (RIL) and Pima populations tested under two irrigation regimes, water-limited (WL) and well-watered (WW) conditions.

**Trait**	**Year**	**Irrigation regime**	**Upland**				**Pima**			
			**Reference thickness**	**THK**	**NDVI**	**SPAD**	**Reference thickness**	**THK**	**NDVI**	**SPAD**
NDVI	2010	WW	−0.13	−0.07	–	0.11	−0.42^*^	−0.73^**^	–	0.06
		WL	−0.18	−0.19	–	−0.07	−0.37^*^	−0.64^**^	–	−0.15
	2011	WW	−0.15	−0.15	–	−0.22^*^	−0.11	−0.15	–	0.06
		WL	−0.03	−0.05	–	−0.20^*^	−0.05	−0.49^*^	–	−0.19
	2012	WW	−0.07	−0.12	–	−0.17	−0.12	0.17	–	0.01
		WL	−0.08	−0.13	–	−0.26^*^	−0.14	−0.58^*^	–	−0.11
Chl_a	2010	WW	0.17	0.14	0.07	0.43^**^	0.30	0.30	−0.17	0.42^*^
		WL	0.24^*^	0.14	−0.21	0.40^**^	0.44^*^	0.30	−0.27	0.41^*^
	2011	WW	0.32^**^	0.17	0.03	0.31^**^	0.35	0.38	−0.37	0.54^**^
		WL	0.11	0.04	0.02	0.30^**^	0.39	0.33	−0.25	0.22
	2012	WW	0.30^**^	0.35^**^	−0.41^**^	0.61^**^	0.33	0.24	−0.34	0.64^**^
		WL	0.35^**^	0.09	−0.10	0.30^**^	0.48^*^	0.40^*^	−0.12	0.36
Chl_ab	2010	WW	0.15	0.12	0.05	0.44^**^	0.34	0.38	−0.28	0.41^*^
		WL	0.23^*^	0.13	−0.20^*^	0.40^**^	0.46^*^	0.38	−0.33	0.41^*^
	2011	WW	0.32^**^	0.18	0.02	0.34^**^	0.36	0.39	−0.35	0.56^**^
		WL	0.08	0.01	−0.01	0.32^**^	0.41^*^	0.35	−0.26	0.25
	2012	WW	0.31^**^	0.36^**^	−0.40^**^	0.60^**^	0.34	0.22	−0.28	0.66^**^
		WL	0.31^**^	0.08	−0.09	0.32^**^	0.49^*^	0.33	−0.05	0.37
SPAD	2010	WW	0.16	0.24^**^	0.09	–	0.10	−0.03	0.06	–
		WL	0.07	0.21^*^	−0.06	–	0.48^*^	0.14	−0.15	–
	2011	WW	0.21^*^	0.16	−0.23^*^	–	0.40^*^	0.28	0.06	–
		WL	0.00	−0.04	−0.20^*^	–	0.11	0.56^**^	−0.19	–
	2012	WW	0.24^**^	0.36^**^	−0.17	–	0.14	0.25	0.01	–
		WL	0.14	0.13	−0.24^*^	–	0.00	0.29	−0.11	–
CID	2010	WW	−0.22^*^	−0.11	−0.13	−0.11	−0.51^*^	−0.69^**^	0.81^**^	−0.05
		WL	−0.15	0.11	0.06	0.02	−0.39	−0.61^**^	0.45^*^	−0.02
	2011	WW	−0.18	−0.10	0.16	−0.19	−0.56^*^	−0.31	−0.30	−0.07
		WL	−0.08	−0.27^**^	0.07	−0.05	−0.16	0.18	−0.33	0.17
	2012	WW	−0.09	−0.17	0.25^*^	0.01	−0.42^*^	−0.15	−0.38	−0.20
		WL	−0.16	−0.04	0.09	−0.17	−0.41^*^	0.42^*^	−0.38	−0.02
D15N	2011	WW	0.19	0.15	−0.14	−0.12	−0.22	−0.16	−0.52^**^	0.06
		WL	0.16	0.03	−0.01	−0.12	0.17	−0.20	0.26	−0.17
	2012	WW	0.20^*^	0.28^**^	−0.20^*^	0.09	0.12	0.01	0.43^*^	−0.13
		WL	0.18	0.11	−0.11	0.08	0.08	−0.14	0.21	−0.29

*Field trials were conducted in 2010–2012 at the Maricopa Agricultural Center located in Maricopa, AZ*.

*, ** *indicate correlations are significant at the P < 0.05 and P < 0.01 levels, respectively*.

The genotypic correlations estimated for the RIL population provided insight into the potential genetic relationship among traits. Under the WW conditions, leaf thickness exhibited significant genotypic correlations with chlorophyll content, both *a* and *ab*, D15N, and CID (${\hat{r}}_{gij}$ values ranging from −0.32 to 0.49, *P* < 0.05 to 0.01, Table 5); these same pairwise trait correlations were not significant under the WL regime. The contrast between treatments is not unexpected given the significant genotype-by-irrigation effect detected for leaf thickness (Table 2). The effect of the irrigation regime on genetic correlations was also evident for two other trait-pairs, namely NDVI/D15N and NDVI/SPAD. For SPAD, the genotypic correlation was only significant under the WL regime whereas for NDVI with D15N, the correlation was only significant in the WW conditions but its value, −0.69, was three times that of the value for the WL conditions, −0.23.

**Table 5:** Genotypic (${\hat{r}}_{gij}$) correlations with standard errors, in parenthesis, and significance levels for the traits evaluated in the upland recombinant inbred line (RIL) population evaluated under water-limited (WL; above the diagonal) and well-watered (WW; below the diagonal) irrigation regimes.

	**NDVI**	**Chl_a**	**Chl_ab**	**D15N**	**CID**	**SPAD**	**THK**	**UHM**	**STR**	**ELO**
**NDVI**		−0.44 (0.16)^**^	−0.42 (0.16)^**^	−0.23 (0.31)^NS^	0.11 (0.16)^NS^	−0.28 (0.14)^*^	−0.25 (0.18)^NS^	−0.22 (0.13)^NS^	−0.01 (0.13)^NS^	0.19 (0.13)^NS^
**Chl_a**	−0.35 (0.16)^*^		0.99 (0.00)^***^	−0.01 (0.32)^NS^	−0.47 (0.16)^**^	0.78 (0.11)^***^	0.23 (0.19)^NS^	0.04 (0.14)^NS^	−0.19 (0.14)^NS^	0.12 (0.14)^NS^
**Chl_ab**	−0.38 (0.15)^*^	0.99 (0.00)^***^		0.04 (.32) ^NS^	−0.49 (0.16)^**^	0.80 (0.10)^***^	0.18 (0.19)^NS^	0.03 (0.14)^NS^	−0.19 (0.13)^NS^	0.14 (0.13)^NS^
**D15N**	−0.69 (0.19)^***^	0.22 (0.20)^NS^	0.21 (0.20)^NS^		−0.22 (0.32)^NS^	0.04 (0.27)^NS^	0.22 (0.36)^NS^	−0.39 (0.26)^NS^	−0.21 (0.24)^NS^	0.10 (0.24)^NS^
**CID**	0.36 (0.16)^*^	−0.42 (0.15)^**^	−0.42 (0.14)^**^	−0.47 (0.20)^*^		0.03 (0.15)^NS^	0.00 (0.19)^NS^	−0.27 (0.13)^*^	−0.30 (0.13)^*^	0.02 (0.13)^NS^
**SPAD**	−0.24 (0.15)^NS^	0.88 (0.08)^***^	0.89 (0.08)^***^	0.14 (0.19)^NS^	−0.18 (0.14)^NS^		−0.01 (0.17)^NS^	0.15 (0.12)^NS^	−0.01 (0.12)^NS^	0.19 (0.11)^NS^
**THK**	−0.20 (0.16)^NS^	0.49 (0.14)^***^	0.46 (0.14)^**^	0.40 (0.20)^*^	−0.32 (0.16)^*^	0.23 (0.14)^NS^		0.04 (.015)^NS^	0.14 (0.15)^NS^	−0.11 (0.15)^NS^
**UHM**	−0.05 (0.13)^NS^	0.17 (0.13)^NS^	0.15 (0.13)^NS^	−0.30 (0.16)^NS^	−0.08 (0.13)^NS^	0.05 (0.12)^NS^	−0.14 (0.13)^NS^		0.53 (0.08)^***^	−0.36 (0.09) ^***^
**STR**	0.09 (0.14)^NS^	−0.03 (0.13)^NS^	−0.05 (0.13)^NS^	−0.17 (0.17)^NS^	−0.08 (0.13)^NS^	−0.07 (0.12)^NS^	0.13 (0.13)^NS^	0.56 (0.08)^***^		−0.25 (0.10) ^*^
**ELO**	0.20 (0.13)^NS^	−0.10 (0.13)^NS^	−0.06 (0.13)^NS^	−0.14 (0.16)^NS^	0.07 (0.13)^NS^	0.10 (0.11)^NS^	−0.10 (0.13)^NS^	−0.35 (0.09)^***^	−0.34 (0.10)^***^	

NS *Not Significant at the < 0.05 level*.

* *Significant at the < 0.05 level*.

** *Significant at the < 0.01 level*.

*** *Significant at the < 0.001 level*.

**Figure 5:**
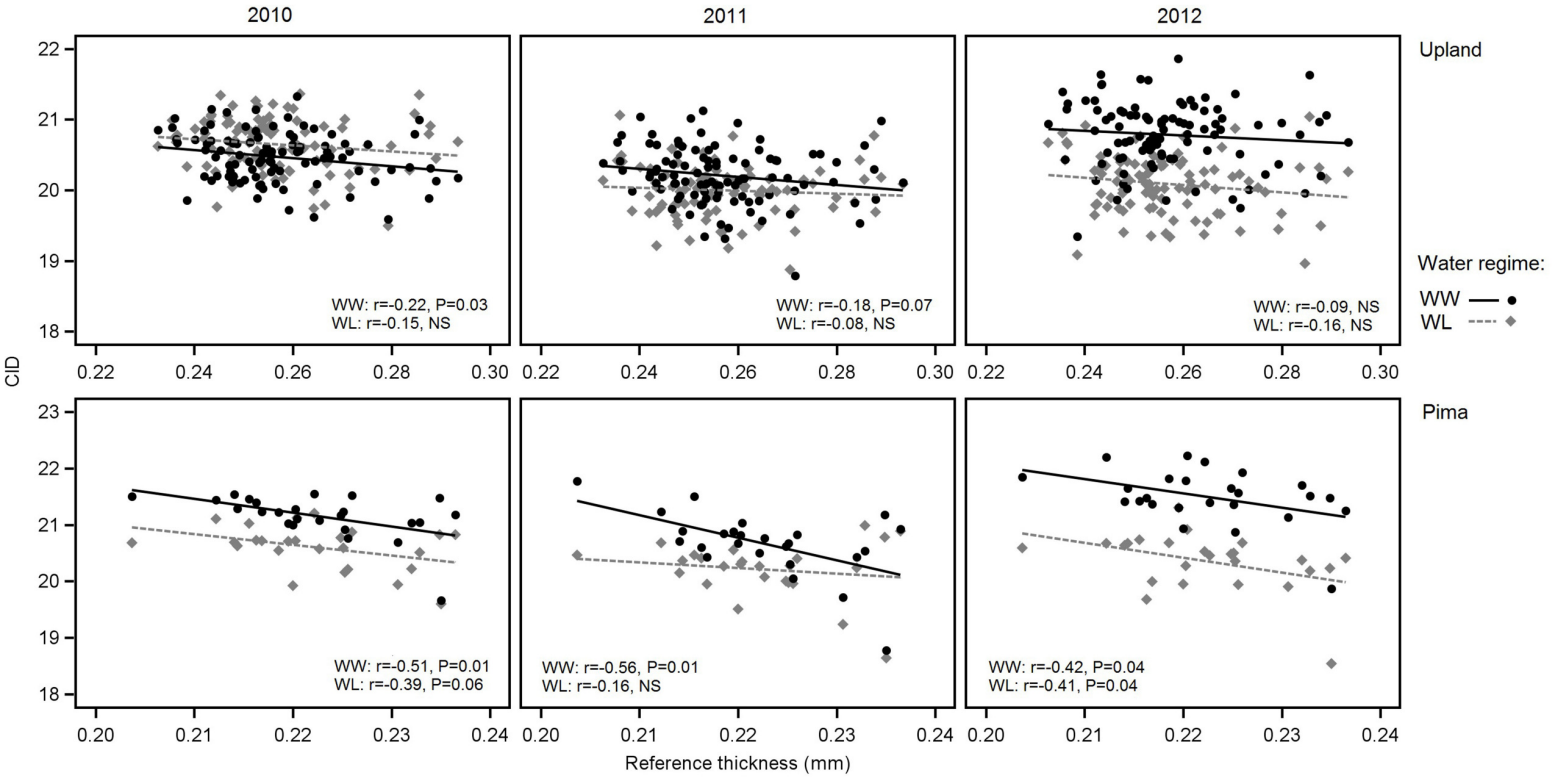
Variation in carbon isotope discrimination (CID) in relation to reference leaf thickness for 2010, 2011 and 2012 and the two irrigation regimes. The upper three graphs are for upland RILs, and the lower three are for the Pima diversity panel. Lines indicate regression trends for each irrigation regime. Note difference in scales for upland vs. Pima graphs.

Consistent with the expectation that thicker leaves are associated with increased water use efficiency, and hence lower CID, the overall trend was that CID decreased with increasing leaf thickness (Table 4; Figure 5). This negative relationship between CID and thickness was also observed in the genetic correlations under WW conditions (Table 5). For the upland population only the correlation in 2010 under WW conditions was significant (*r* = −0.22, *P* < 0.05) between reference leaf thickness and CID. However, for the Pima population four of the six possible correlations between reference leaf thickness and CID were significant (*P* < 0.05) with correlation values (*r*) ranging from −0.41 to −0.56; three of those significant correlations were observed under WW conditions. Otherwise, CID showed no consistent phenotypic trends with NDVI or SPAD values (Table 4). However, CID did display significant genetic correlations with NDVI under WW conditions as well as chlorophyll *a* and *ab* under both irrigation regimes.

In assessing possible relationships between leaf thickness and fiber quality, neither the upland nor the Pima populations showed effects of either reference leaf thickness or single-season/treatment thickness values (Supplementary Figure 1; Supplementary Table 4). However, when assessing the relationship of fiber quality with NDVI and SPAD values, the two populations exhibited markedly different characteristics. The Pima fiber quality traits all had significant, negative correlations with NDVI, and with regard to SPAD, fiber length and strength had significant, negative correlations; the upland population exhibited correlations close to zero for these associations (Supplementary Table 4).

Given the effects of year and irrigation regime on crop traits (Table 2), multiple linear regression was used to estimate whether variation in key traits was explained by the reference leaf thickness once mean effects of irrigation regime and year were considered (Table 6). For NDVI in the upland RIL population, variation in reference leaf thickness explained only 1% of the residual sums of squares whereas for the Pima population, reference leaf thickness explained a significant (*P* = 0.01) amount, 5%, of the residual variance. For chlorophyll *a*, reference thickness had a much more significant effect (*P* < 0.001) on the trait; it explained 10 and 8.7% of the residual trait variance for the RIL and Pima populations, respectively. The trait that exhibited the largest difference between populations with respect to the portion of variance explained by leaf thickness was CID. Leaf thickness explained over 17% of the variation in CID in contrast to only accounting for ~3% in the RIL population. Combined, these results further support the conclusion that leaf thickness contributes to the variation observed in leaf physiological traits.

**Table 6:** Analysis of variance (ANOVA) for multiple regressions that test for influence of reference leaf thickness on NDVI, chlorophyll *a* concentration, SPAD, and carbon isotope discrimination (CID) once effects of irrigation regime within years are considered. Thus, tests are for sequential (Type I) sums of squares (SS).

**Trait**	**Population**	**Source**	**DF**	**Type I SS**	**Mean Square**	***F*-value**	**Probability for F**	**Residual SS (%)**
NDVI	Upland	I(Y)	5	1.91	0.38	132.6	<0.001	
		Ref. Leaf thickness	1	0.02	0.02	6.1	<0.050	1.1
		Residual	575	1.66				
	Pima	I(Y)	5	0.85	0.17	46.8	<0.001	
		Ref. Leaf thickness	1	0.03	0.03	7.6	<0.010	5.0
		Residual	143	0.52				
Chl_a	Upland	I(Y)	5	2,279.72	455.94	129.3	<0.001	
		Ref. Leaf thickness	1	238.22	238.22	67.6	<0.001	10.0
		Residual	611	2,154.82				
	Pima	I(Y)	5	2,009.05	401.81	158.5	<0.001	
		Ref. Leaf thickness	1	34.73	34.73	13.7	<0.001	8.7
		Residual	143	362.45				
SPAD	Upland	I(Y)	5	1,603.70	320.74	57.8	<0.001	
		Ref. Leaf thickness	1	62.42	62.42	11.2	<0.001	1.9
		Residual	581	3,191.71				
	Pima	I(Y)	5	785.08	157.02	51.3	<0.001	
		Ref. Leaf thickness	1	16.59	16.59	5.4	<0.050	3.7
		Residual	143	437.61				
CID	Upland	I(Y)	5	19.27	3.85	40.0	<0.001	
		Ref. Leaf thickness	1	2.07	2.07	21.5	<0.001	3.4
		Residual	611	58.80				
	Pima	I(Y)	5	24.70	4.94	31.3	<0.001	
		Ref. Leaf thickness	1	4.85	4.85	30.8	<0.001	17.7
		Residual	143	22.54				

*I(Y) represents the model term irrigation regime nested within year*.

Finally, the QTL analysis revealed four unique genomic locations, on chromosomes D02, D03, D08, and D09, responsible for the variation in leaf thickness (Table 7). The detected QTL on D09 was identified under both irrigation regimes, and on average, explained 13.40% of the observed variation. Of the remaining identified QTL, which were all detected in the WL irrigation regime, the one located on D08 explained the largest portion of phenotypic variation at 18.58% and had an effect estimate of 0.006 mm.

**Table 7:** Summary of quantitative trait loci (QTL), detected at an experiment-wise Type I error rate of 5%, for leaf thickness in the upland recombinant inbred line (RIL) population. The RIL population was evaluated under water-limited (WL) and well-watered (WW) conditions in 2010–2012.

**Irrigation regime**	**Chr.^a^**	**Linkage group**	**Peak position**	**Left marker**	**Left marker position**	**Right marker**	**Right marker position**	**LOD^b^**	**PVE^c^**	**Allelic effect^d^**
WL	D02	62	7	SNP0043	0.00	SNP0152	8.02	3.76	11.49	−0.005
WL	D03	70	1	DPL0217a	0.00	BNL3590a	4.07	3.98	12.15	0.005
WL	D09	98	35	DPL1130a	33.14	TMB0382a	35.68	3.98	11.96	−0.005
WW	D09	98	35	DPL1130a	33.14	TMB0382a	35.68	4.07	14.83	−0.005
WL	D08	105	9	SNP0005	3.52	SNP0452	9.01	6.04	18.58	−0.006

*Marker positions are reported in centimorgans (cM)*.

a *Chr., chromosome to which the linkage group belongs, based on Pauli et al. (2016a)*.

b *LOD, logarithm of odds*.

c *PVE, percent phenotypic variation explained, percentage*.

d *Allelic effect, effect when substituting a NM24016 allele with an allele from TM-1, in mm*.

## Discussion

Field based HTP allows for the rapid collection of valuable phenotypic data under real-world production conditions, such as heat and drought stress. Central to utilizing these data for crop improvement is understanding how basic morphometric properties of the plant canopy impact radiometric properties. This knowledge will be critical as the plant science community transitions into working with larger genetic populations such as the planned 5,000 line upland cotton nested association mapping (NAM) panel and the currently in-development *G. barbadense* diversity panel of ~400 lines (White et al., 2012; Hinze et al., 2016). However, before these larger populations can be leveraged to their full extent, a foundational knowledge of leaf properties must be developed in order to account for the effects when larger-scale phenotyping projects are initiated; these larger populations represent a much more complex genetic system. To address this knowledge gap, we undertook the present study using tractable experimental populations of 95 upland RILs and a modest sized collection of 25 Pima cultivars. These panels were selected because of their past characterization, and with respect to the RIL population, serve as a benchmark resource within the cotton genetics community (Gore et al., 2012, 2014; Andrade-Sanchez et al., 2014; Fang et al., 2014; Thorp et al., 2015). We evaluated both populations under contrasting irrigation regimes to assess the effects of leaf thickness on spectral reflectance measured using HTP methods. The relationships between leaf thickness and other physiological and fiber quality traits were also assessed to identify potential shared biology resulting from simple variation in leaf thickness.

The upland (*G. hirsutum*) and Pima (*G. barbadense*) populations both exhibited variation for leaf thickness, and broad-sense heritabilities were generally high regardless of irrigation regime (Table 1, Figure 2). This finding, in combination with the QTL identified in the upland RIL population, provides further evidence that leaf thickness is a trait with a strong genetic basis in cotton. With respect to the actual leaf thicknesses, the upland RILs consistently had thicker leaves than the Pima lines, on average 0.035 mm thicker. Although the main effect of irrigation regime was nonsignificant for the two populations studied, the interaction effects of genotype-by-irrigation regime and genotype-by-year were highly significant confirming that genotypes from both species responded differentially to growing conditions. This can be exemplified by the decline in thickness for the Pima population in 2012 relative to 2010 and 2011 (Figure 2). In 2012, due to a period of rainy weather (Figure 1), thickness measurements were delayed which may have permitted new leaves to emerge. If these new leaves were formed under lower irradiance conditions, they would be expected to be thinner (Patterson et al., 1977; Evans and Poorter, 2001), which suggests that leaf thickness of Pima germplasm may be sensitive to prior weather or management on a time scale of a few weeks.

Several apparent differences between the upland and Pima populations highlight the diversity in genetic composition and the consequences that diversity can have on phenotypic relationships. With respect to effect of the irrigation regime on all traits other than leaf thickness, a stark contrast is observed between the two populations; excluding leaf thickness, eight out of the 10 traits for the Pima population showed highly significant (*P* < 0.01) irrigation regime effects in contrast to the upland population where only two traits were significant for irrigation. This observation, in combination with the differences in correlation values for NDVI and leaf thickness, as well as the higher heritability estimates for the Pima population (one-sided *t*-test, *P* < 0.01), highlight the different genetic structures of the two germplasm assemblages. The upland population only captures the genetic variation present in just two parental genotypes whereas the Pima population is composed of genotypes representing 90 years of breeding and selection. Because of this difference in population composition, there is more genetic and allelic variation present in the Pima population that likely impacts the differences in phenotypic variation as well as response to water deficit (Falconer and Mackay, 1996). These genetic and phenotypic differences are further supported by the developmental history of American Pima lines which involved the intercrossing of germplasm from various geographical regions, including germplasm of Peruvian and Sea Island descent (Peebles, 1954; Feaster and Turcotte, 1962; Smith et al., 1999; Percy, 2009).

However, there is an associated limitation in using a diverse panel of Pima lines that span a time continuum and capture more genotypic and phenotypic diversity than that of a biparental population. The statistically significant correlations observed between NDVI and fiber quality traits in the Pima population must be carefully interpreted as they are confounded by breeding history and overall plant improvement. The earliest released lines had low leaf/stem biomass yield but these characteristics progressively increased over time due to selection for plant productivity along with simultaneous genetic improvements to stress tolerance (or avoidance), yield, and fiber quality. Further compounding the issue of trait correlations is the relatedness among the lines themselves as superior genotypes (those lines that were released for commercial production) or close relatives were likely used as parents for the next cycle of breeding. Without molecular marker data or pedigree information, we were unable to account for this relatedness in our analyses, an area of potential improvement in our current work because line relatedness and year of release could impact other correlations as well. Correlations between NDVI and fiber quality traits were nonexistent in the upland population. Such a lack of association is likely due to having two mostly modern parental genotypes as population founders and a population mating design that reshuffled parental genomes by recent recombination during RIL development. Taken together, this essentially negated the issues of release date and population structure.

Despite these differences in genetic structure between the two populations, the observed contrasts in the physical properties of the plants themselves are still likely due to underlying physiological differences for abiotic stress tolerance between the two species (Dabbert and Gore, 2014). Upland cotton is generally considered better adapted to drought given its Mesoamerican origin compared with Pima which originated from northwest South America near bodies of water (Saranga et al., 2004; Wendel et al., 2010). Because of their divergent origins, both species may have evolved different methods for environmental adaptions to stress environments like those conditions found in our study (Saranga et al., 1998). This contrast in adaptive ability is further supported by Saranga et al. (2004) who found that there was contrasting loci with favorable allelic variation in either species for stress-adaptive traits. Evidence of this nature provides some insight into how these two species respond to environmental conditions and give rise to the observed differences between the species and populations used.

Correlations between leaf thickness and NDVI for the upland population were low in contrast to the Pima population, which had strong, negative correlations between the two traits. For the Pima lines, NDVI decreased with greater thickness (Table 4), which is consistent with the expectation that thicker leaves may be associated with reduced leaf area and hence NDVI. This result raises the question about the utility of using NDVI, or more generally spectral reflectance data, as a selection tool for leaf thickness. Previous laboratory-based analyses using passive hyperspectral sensors with individual leaves have detected strong correlations between leaf thickness and NIR reflectance (wavelengths ranging from 750 to 1,350 nm) in cotton (Zhang et al., 2012) as well as diverse species (Knapp and Carter, 1998; Seelig et al., 2008). In comparison, our study utilized an active, multispectral radiometer with only one NIR band (820 nm) measuring canopy-level reflectance in the field. Our field-based, canopy-level results suggest that if there is an appreciable amount of phenotypic variation, such as in an association mapping panel or a diverse collection of elite cultivars, NDVI could potentially be a useful selection tool for leaf thickness. However, NDVI measurements within breeding families, like the RIL population used in this study, may not adequately discriminate leaf thickness amongst related lines given the low correlation values we observed. To extend this work, further research is needed to exclude alternate factors such as differences in canopy architecture or leaf anatomy, including possible gene pool differences in leaf thickness as found in common bean (*Phaseolus vulagris* L.) (Sexton et al., 1997), to better understand the dynamics of NDVI as related to leaf thickness. Overall, the trends with NDVI support our proposition that FB-HTP involving canopy reflectance measurements should consider phenotypic variation in leaf thickness as an underlying cause of variation in NDVI with potentially large effects on other physiological traits.

The correlations between leaf thickness and other leaf traits were consistent with the expectation that thicker leaves would have a greater chlorophyll concentrations and hence SPAD readings. Weak negative correlations with CID agreed with previous research where genotypes with thicker leaves had greater transpiration efficiency (Rao and Wright, 1994; Rebetzke et al., 2008). This assessment is further supported by the genetic correlation analyses carried out in the RIL population. The genetic correlations revealed a significant negative relationship between leaf thickness and CID and positive correlations with chlorophyll content (both chlorophyll *a* and *ab*) and D15N under WW conditions. This finding suggests a shared genetic basis between leaf thickness and these physiological traits, and furthermore, emphasizes the value in understanding how genetic variation in cotton leaf thickness affects fundamental physiological crop traits. In contrast, the lack of phenotypic and genotypic associations between leaf thickness and fiber quality parameters (Table 5, Supplementary Table 3) suggest that selection directly affecting leaf thickness would not affect fiber quality through possible developmental correlations.

After accounting for the effect of irrigation, the use of a reference leaf thickness value (a derived trait representing the idealized phenotype not confounded by environmental effects) for linear regression provided a means to assess the impact of leaf thickness on other canopy component traits. Although percent variation explained by reference thickness was low, which may be due to the shortcoming of using a reference value based on only 3 years of data, the estimated portions of variance were still significant, especially for the traits chlorophyll *a* and CID. These results demonstrate how physical characteristics impact both the radiance and physiological properties of leaves. Given these findings in combination with the strong genetic basis of leaf thickness, supported by the relatively moderate to high heritability estimates and the detection of loci controlling phenotypic variability, it is clear that further investigation of this trait is warranted. Selection on leaf thickness itself, which should respond quite favorably, could be beneficial in producing more stress resilient cotton plants that are able to better maintain key fiber quality traits when faced with environmental challenges. The use of molecular markers in linkage with causal loci for leaf thickness, like those identified herein, could further aid in the selection of plants with desirable leaf characteristics. However, an unresolved issue is whether leaf thickness is best measured manually, as done here, or can be related to data from proximal or remote sensing either through direct associations with specific reflectance indices or via inversion of a radiative transfer model (Thorp et al., 2015).

## Conclusion

Measuring the thickness of cotton leaves with a micrometer allowed for reliable non-destructive sampling that identified large genetic differences for both upland and Pima cotton populations. The Pima lines showed potential relations with NDVI that support a tradeoff between thicker leaves and reduced canopy development and suggest a potential confounding factor in using canopy reflectance in FB-HTP. Leaf thickness also affected CID, more so in the Pima population where a greater proportion of significant correlations were observed than in the upland population, implying a direct effect on leaf-level transpiration efficiency. However, variation in thickness was not associated with fiber quality. Line-by-year and line-by-irrigation regime interactions emphasize the need to understand how leaf thickness might vary with in-season environmental conditions, especially in large-scale phenotyping efforts. Overall, our results support considering variation in leaf thickness as a potential contributing factor to variation in NDVI or other traits measured via proximal or remote sensing and as a trait that impacts other physiological responses.

## Author contributions

JW and MG conceived the experimental design; JW, PA, MC, JH, KT, AF, DH, EC, GW, and MG collected phenotypic data; DP, JW, and MG conceptualized the analysis; DP and JW performed the analyses and wrote the manuscript; DP, JW, MG, KT, AF, EC, MC, PA, and GW revised the manuscript.

### Conflict of interest statement

The authors declare that the research was conducted in the absence of any commercial or financial relationships that could be construed as a potential conflict of interest.
