# Supplementary material for: Investigation of the Influence of Leaf Thickness on Canopy Reflectance and Physiological Traits in Upland and Pima Cotton Populations
Source: Front Plant Sci. 2017 Aug 17;8:1405. doi: 10.3389/fpls.2017.01405 (PMC5563404; doi:10.3389/fpls.2017.01405)
Supplement: Supplementary file 1 [file Table1.PDF]

Supplementary Table 1. Summary of crop calendars including timing of key field phenotyping activities.

Date(s)	Activity
2010	
7 May	Planting
13 July	Water-limited irrigation regime started
27-30 July	Leaf thickness measured and specific leaf weight sampling
29 July	SPAD readings taken
5 August	Spectral reflectance (NDVI) measured
19 August	Leaf disks for chlorophyll, $\delta^{13}C$ and $\delta^{15}N$
8 October	Defoliant applied
2011	
21 April	Planting
8 July	Water-limited irrigation regime started
2, 8 August	SPAD readings taken
3, 4, 8 August	Leaf thickness measured
4 August	Spectral reflectance (NDVI) measured
8 September	Leaf disks for chlorophyll, $\delta^{13}C$ and $\delta^{15}N$ collected
23 September	Defoliant applied
2012	
26 April	Planting
18 June	Water-limited irrigation regime started
28-29 August	SPAD readings taken
29 August - 5 September	Leaf thickness measured
30 August	Spectral reflectance (NDVI) measured
5 September	Leaf disks for chlorophyll, $\delta^{13}C$ and $\delta^{15}N$ collected
27 September	Defoliant applied
