# Supplementary material for: Investigation of the Influence of Leaf Thickness on Canopy Reflectance and Physiological Traits in Upland and Pima Cotton Populations
Source: Front Plant Sci. 2017 Aug 17;8:1405. doi: 10.3389/fpls.2017.01405 (PMC5563404; doi:10.3389/fpls.2017.01405)
Supplement: Supplementary file 2 [file Table2.PDF]

Supplementary Table 2. Mean, minimum, maximum, and standard deviation of best linear unbiased estimators (BLUEs) for fiber quality traits evaluated for the upland recombinant inbred line (RIL) and Pima populations tested under two irrigation regimes, water-limited (WL) and well-watered (WW) conditions. Estimates of broad-sense heritability ($\hat{H}^2$) are on an entry mean basis. Field trials were conducted from 2010 to 2012 at the Maricopa Agricultural Center located in Maricopa, AZ.

			**RILs**						**Pima**					
**Trait**	**Year**	**Irrigation regime**	**Mean**	**Min**	**Max**	**SD**	**($\hat{H}^2$)**	**SE of ($\hat{H}^2$)**	**Mean**	**Min**	**Max**	**SD**	**($\hat{H}^2$)**	**SE of ($\hat{H}^2$)**
Upper half mean (mm)	2010	WL	29.46	25.65	32.77	1.52	0.93	0.02	34.80	33.02	36.58	1.02	0.90	0.04
		WW	29.46	25.65	33.27	1.52	0.88	0.02	34.80	32.77	36.32	1.02	0.88	0.04
	2011	WL	28.45	24.89	31.24	1.52	0.95	0.01	35.81	33.27	38.10	1.27	0.94	0.02
		WW	28.45	24.64	31.50	1.52	0.95	0.01	36.32	34.04	39.12	1.27	0.93	0.03
	2012	WL	28.45	24.38	31.24	1.27	0.91	0.02	35.56	33.53	37.59	1.02	0.90	0.03
		WW	28.96	24.64	32.51	1.52	0.94	0.01	36.83	34.04	39.37	1.27	0.88	0.04
Fiber strength (kN m kg^{-1})	2010	WL	33.72	28.48	40.84	2.60	0.91	0.02	42.70	36.77	48.87	3.23	0.94	0.02
		WW	33.31	28.97	38.81	2.29	0.85	0.03	41.83	37.15	49.05	3.12	0.94	0.02
	2011	WL	32.02	26.52	37.63	2.44	0.93	0.02	42.26	37.07	46.93	3.16	0.87	0.04
		WW	31.41	26.22	36.67	2.32	0.89	0.02	42.61	35.63	50.40	3.47	0.90	0.04
	2012	WL	32.61	28.04	37.92	2.46	0.91	0.02	42.67	37.80	48.73	3.33	0.90	0.03
		WW	33.12	28.56	39.04	2.47	0.91	0.02	43.68	36.20	50.03	3.76	0.89	0.04
Fiber elongation (%)	2010	WL	5.14	3.16	7.62	0.86	0.96	0.01	6.14	5.54	7.27	0.43	0.90	0.04
		WW	5.21	3.26	7.03	0.88	0.95	0.01	5.95	5.35	6.96	0.43	0.88	0.04
	2011	WL	5.33	3.52	7.24	0.76	0.96	0.01	5.58	4.68	6.38	0.38	0.89	0.04
		WW	5.26	3.41	7.37	0.72	0.96	0.01	5.53	4.69	6.36	0.39	0.81	0.07
	2012	WL	4.70	2.87	6.27	0.76	0.96	0.01	7.14	6.31	7.98	0.47	0.86	0.05
		WW	4.83	2.85	6.71	0.80	0.95	0.01	7.10	6.38	8.14	0.50	0.89	0.04
