# Supplementary material for: Investigation of the Influence of Leaf Thickness on Canopy Reflectance and Physiological Traits in Upland and Pima Cotton Populations
Source: Front Plant Sci. 2017 Aug 17;8:1405. doi: 10.3389/fpls.2017.01405 (PMC5563404; doi:10.3389/fpls.2017.01405)
Supplement: Supplementary file 3 [file Table3.PDF]

Supplementary Table 3. Correlation of specific leaf weights calculated on fresh (SLW_{fr}) and dry weight (SLW_{dr}) bases with actual (in season) and reference leaf thickness (Reference) for the upland and Pima populations in 2010 under well-watered (WW) and water-limited (WL) conditions.

		Upland		Pima	
	Irrigation regime	Actual	Reference	Actual	Reference
SLW_{fr}	WW	0.10	0.14	0.69**	0.49*
	WL	0.40**	0.26*	0.73**	0.50*
SLW_{dr}	WW	0.02	0.04	0.32	0.24
	WL	0.36**	0.24*	0.28	0.18

*, ** Indicate correlations are significant at the $P < 0.05$ and $P < 0.01$ levels, respectively
