# Supplementary material for: Investigation of the Influence of Leaf Thickness on Canopy Reflectance and Physiological Traits in Upland and Pima Cotton Populations
Source: Front Plant Sci. 2017 Aug 17;8:1405. doi: 10.3389/fpls.2017.01405 (PMC5563404; doi:10.3389/fpls.2017.01405)
Supplement: Supplementary file 4 [file Table4.PDF]

Supplementary Table 4. Phenotypic correlations (Pearson's) estimated among various leaf and fiber quality traits for the upland recombinant inbred line (RIL) and Pima populations tested under two irrigation regimes, water-limited (WL) and well-watered (WW) conditions. Field trials were conducted in 2010 - 2012 at the Maricopa Agricultural Center located in Maricopa, AZ.

			Upland				Pima			
**Trait**	**Year**	**Irrigation regime**	**Reference thickness**	**THK**	**NDVI**	**SPAD**	**Reference thickness**	**THK**	**NDVI**	**SPAD**
Upper half mean (mm)	2010	WW	-0.10	-0.15	0.07	0.06	-0.08	-0.00	-0.47*	-0.34
		WL	-0.08	-0.06	-0.09	0.11	-0.21	-0.34	-0.38	-0.47*
	2011	WW	-0.11	-0.07	0.00	0.07	-0.23	-0.14	-0.27	-0.35
		WL	-0.07	0.01	0.03	0.13	-0.01	-0.06	-0.30	-0.57**
	2012	WW	-0.11	-0.07	0.09	0.04	-0.09	-0.16	0.16	-0.26
		WL	-0.06	0.16	-0.12	0.05	-0.15	-0.24	0.29	-0.57**
Fiber strength (kN m kg^{-1})	2010	WW	0.16	0.09	0.08	0.01	-0.32	-0.10	-0.11	-0.12
		WL	0.11	0.06	0.07	-0.03	-0.31	-0.35	-0.26	-0.26
	2011	WW	0.09	0.02	0.08	0.02	-0.19	-0.04	-0.52**	-0.24
		WL	0.10	0.10	-0.06	0.03	-0.12	0.02	-0.57**	-0.29
	2012	WW	0.08	0.16	0.09	0.00	-0.17	-0.24	-0.46*	0.18
		WL	0.07	0.13	0.11	-0.01	-0.21	0.23	-0.28	-0.04
Fiber elongation (%)	2010	WW	-0.06	-0.01	0.09	0.08	0.04	0.11	0.44*	-0.03
		WL	-0.06	-0.01	0.14	0.06	0.06	0.31	0.48*	0.22
	2011	WW	-0.08	-0.01	0.12	0.12	0.15	-0.01	0.37	-0.08
		WL	-0.04	-0.09	0.04	0.16	0.12	-0.04	0.20	-0.05
	2012	WW	-0.12	-0.18	0.09	0.01	0.02	0.11	0.18	-0.30
		WL	-0.13	-0.10	0.22*	0.19	0.14	-0.24	0.22	-0.03

*, ** Indicate correlations are significant at the $P < 0.05$ and $P < 0.01$ levels, respectively.
