# Supplementary material for: Investigation of the Influence of Leaf Thickness on Canopy Reflectance and Physiological Traits in Upland and Pima Cotton Populations
Source: Front Plant Sci. 2017 Aug 17;8:1405. doi: 10.3389/fpls.2017.01405 (PMC5563404; doi:10.3389/fpls.2017.01405)
Supplement: Supplementary file 5 [file Image1.PDF]

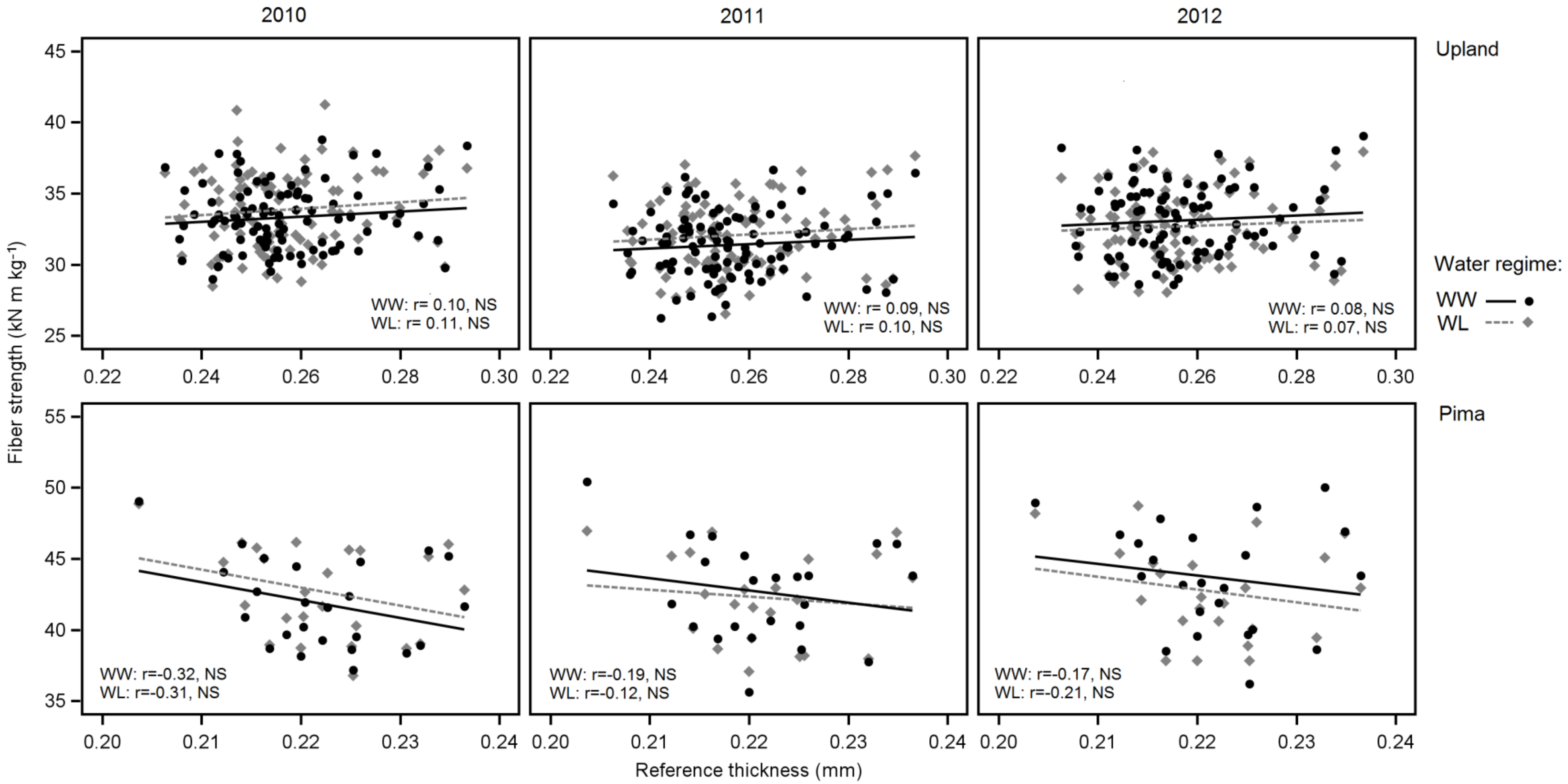

Supplementary Figure 1. Variation in cotton fiber strength in relation to reference leaf thickness for 2010, 2011 and 2012 and the two irrigation regimes. Upper three graphs are for upland RILs and lower three are for the Pima diversity panel. Lines indicate regression trends for each irrigation regime. Note difference in scales for upland vs. Pima graphs.
